# Cardioprotective effects of exercise training on doxorubicin-induced cardiomyopathy: a systematic review with meta-analysis of preclinical studies

**DOI:** 10.1038/s41598-021-83877-8

**Published:** 2021-03-18

**Authors:** Paola Victória da Costa Ghignatti, Laura Jesuíno Nogueira, Alexandre Machado Lehnen, Natalia Motta Leguisamo

**Affiliations:** Post-Graduate Program in Health Sciences: Cardiology, Institute of Cardiology of Rio Grande do Sul/University Foundation of Cardiology, Av. Princesa Isabel, 370, Porto Alegre, Rio Grande do Sul CEP 90620-001 Brazil

**Keywords:** Cancer, Health care

## Abstract

Doxorubicin (DOX)-induced cardiotoxicity in chemotherapy is a major treatment drawback. Clinical trials on the cardioprotective effects of exercise in cancer patients have not yet been published. Thus, we conducted a systematic review and meta-analysis of preclinical studies for to assess the efficacy of exercise training on DOX-induced cardiomyopathy. We included studies with animal models of DOX-induced cardiomyopathy and exercise training from PubMed, Web of Sciences and Scopus databases. The outcome was the mean difference (MD) in fractional shortening (FS, %) assessed by echocardiography between sedentary and trained DOX-treated animals. Trained DOX-treated animals improved 7.40% (95% CI 5.75–9.05, p < 0.001) in FS vs. sedentary animals. Subgroup analyses revealed a superior effect of exercise training execution prior to DOX exposure (MD = 8.20, 95% CI 6.27–10.13, p = 0.010). The assessment of cardiac function up to 10 days after DOX exposure and completion of exercise protocol was also associated with superior effect size in FS (MD = 7.89, 95% CI 6.11–9.67, p = 0.020) *vs.* an echocardiography after over 4 weeks. Modality and duration of exercise, gender and cumulative DOX dose did were not individually associated with changes on FS. Exercise training is a cardioprotective approach in rodent models of DOX-induced cardiomyopathy. Exercise prior to DOX exposure exerts greater effect sizes on FS preservation.

## Introduction

Despite substantial advances in cancer treatment due to development of targeted therapies and immunotherapy, anthracycline-based regimens remain the backbone of several antineoplastic treatments^1^. Doxorubicin (DOX), an anthracycline, is listed as essential medicine by World Health Organization (WHO) and has a prominent role in the management of breast cancer and pediatric hematological malignancies^2^. Unfortunately, left ventricular dysfunction and clinical heart failure in occur in 9% of DOX-treated patients, restricting the use of this drug^3^. While acute DOX-induced cardiovascular disorders may seriously impair the optimal efficacy of antineoplastic regimens and patient’s eligibility to sequential treatments^4^, long-term consequences of DOX exposure raises as a major concern as the population of cancer survivors continues to increase^5,6^. Thus, the search for cardioprotective therapies to prevent or minimize DOX-cardiovascular toxicity is of great interest to ensure both successful anticancer activity and cardiovascular health.

Since early studies reported the association between DOX-induced heart failure and total cumulative dose^7^, the counterintuitive cardioprotective strategy relied on dose reduction of this anthracycline. However, cardiotoxicity was still observed in patients receiving less than recommended cumulative dose^3^. Therefore, in order to maintain the proper DOX doses to meet oncological outcomes accordingly, pharmacological cardioprotective strategies, such as Dexrazoxane and neurohormonal therapy (β-blockers, angiotensin receptor blockers, angiotensin-converting enzyme inhibitors) have been broadly explored. It has been recently reported in two meta-analyses that both strategies have a role in reducing the risk of heart failure and cardiac events in patients exposed to anthracyclines-based regimens^8,9^.

Alternatively, non-pharmacological cardioprotective strategies, such as exercise training, have been under investigation against cardiovascular events in cancer patients. Current evidence on exercise training has demonstrated a significant role in the prevention and rehabilitation of cardiovascular disorders within the antineoplastic-induced cardiotoxicity, and particularly in those cardiotoxic events induced by anthracyclines^10^. Exposure to exercise training before cancer diagnosis is associated with a 20–37% risk reduction of adverse cardiovascular events in patients with primary breast cancer^11^. However, while these evidences demonstrate that regular physical activity in cancer patients improve both oncological and cardiovascular outcomes^11–14^, the existing body of evidence still lacks of randomized clinical trials and high level data with respect to modalities, duration, intensity and ideal timing of exercise realization in relation to the schedule of cancer treatment.

With the purpose of scaling up observational studies with controlled supervised exercise programs and increasing the number of randomized clinical trials, experimental studies are an indispensable source of evidences. Although a significant number of studies exploring the effects of exercise on animal models of DOX-induced cardiomyopathy have been published, as for humans, there is no consensus on the ideal exercise protocol. Hence, we conducted this systematic review with meta-analysis to summarize the available information of the influence of exercise practice in prevention and/or attenuation of DOX-induced cardiotoxicity in rodents.

## Methods

This review agrees with Preferred Reporting Items for Systematic Reviews and Meta-Analyses (PRISMA) guidelines^15^.

### Search strategy

The web search included experimental studies which evaluated the impact of exercise in the prevention or attenuation of DOX-induced cardiotoxicity reviewed in Public Medline (PubMed), Web of Science and Scopus databases. There were no language or publication status limits and all studies from the inception to April 2020 were selected for eligibility assessment. The selection of descriptors considered the practice of physical exercise and its influence on cardiac function of animals treated with anthracyclines. The search was comprehensive with terms or correlational terms and included “exercise” and “doxorubicin” as investigation strategy. The detailed search strategies are available in Supplementary Table 1.

### Inclusion criteria and type of outcome measurements

We included only controlled and comparative studies with rats or mice models of DOX-induced cardiomyopathy who underwent any type of physical activity. The inclusion of the studies required the presence of both DOX-treated sedentary and trained animals. We excluded the studies which had hormonal modulation in DOX-treated animals, comorbidities (diabetes, hypertension or mental preconditions) or combinatory interventions with exercise (restriction diet or supplementation). However, if the study has included DOX-treated sedentary and exercised groups as controls of other variables/interventions, we extracted the data of only of these groups. The main outcome referred specifically to the recording of fractional shortening (FS, %), which must have been assessed by echocardiography.

### Study selection

**Figure 1:**
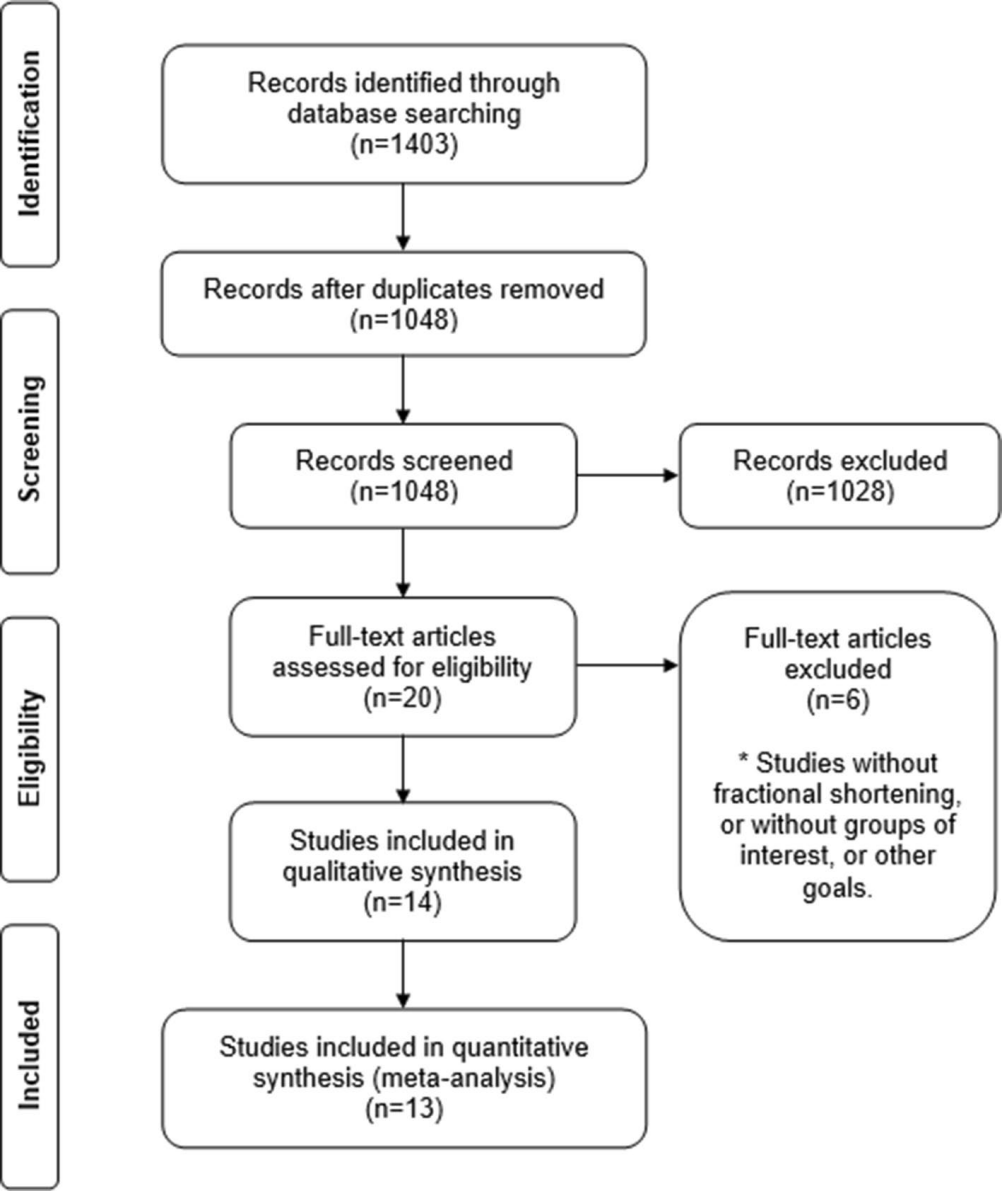
Flow chart of the study selection procedure.

Prior to initial search, no filter was applied and the databases yielded 1403 articles. After duplicates deleting, 1048 articles were selected for analysis and cross-checking by a pair of independent researchers (P.V.C.G. and L.J.N.). Following analysis of titles and abstracts, we excluded 1,028 articles which did not meet the aim of this review, and 5 studies which did not meet the inclusion criteria and/or did not evaluate FS as outcome of cardiac function (Fig. 1).

Finally, 14 studies^16–29^ were critically examined. Next, the following information were retrieved: identification of the manuscript (authors information and publication year); general characteristics of the animals (rat or mice, age/weight and sex); model of cardiotoxicity induction (dose, frequency and administration of DOX); exercise protocol (modality, start point, duration and protocol of training); cardiac function assessment (timepoints and FS mean and standard deviation). Additionally, we extracted key information on exercise and DOX protocols, and risk of bias. When relevant, the correspondent author was contacted if essential information or data were unclear or not-available on the manuscript. If FS mean and standard deviation were not numerically described, we used GetData (GetData Graph Digitizer software, version 2.26) to extract the data from graphs.

Some of the included studies have been designed to investigate the interaction of exercise training or animal model covariables in cardiovascular function of DOX-treated animals (duration, modalities, DOX cumulative dose), and have more than two groups of interest. As for these studies, each experimental group with retrieved data is referred in the forest plots as follows: Hydock et al., 2008^19^ treadmill group (a) at day 5 and (b) at day 10, (c) wheel running group at day 5 and (d) at day 10; Hydock et al., 2011^20^ (a) treadmill group, (b) wheel running group; Hydock et al., 2012^21^ (a) 15 mg/kg and (b) 20 mg/kg cumulative dose of DOX; Jensen et al., 2013^22^ treadmill group (a) at day 1, (b) at day 3, (c) at day 5, (d) at day 7 and (e) at day 9, wheel running group (f) at day 1, (g) at day 3, (h) at day 5, (i) at day 7 and (j) at day 9; Lien et al., 2015^23^ treadmill group receiving (a) 10 mg/kg and (b) 15 mg/kg cumulative dose of DOX, wheel running group receiving (c) 10 mg/kg and (d) 15 mg/kg cumulative dose of DOX; Parry et al., 2015^26^ wheel running group (a) at day 1, (b) at day 3 and (c) at day 5; Wang et al., 2018^29^ mice (a) C57BL/6 and (b) Athymic Swiss Nude.

### Methodological quality and risk of bias

Qualitative analyses were performed by 3 different tools. Systematic Review Center for Laboratory Animal Experimentation’s (SYRCLE)^30^ and Collaborative Approach to Meta-Analysis and Review of Animal Data from Experimental Studies (CAMARADES)^31^, which are a risk of bias tool specifically used for animal experimentation. Review Manager (RevMan software, version 5.3, The Nordic Cochrane Center, The Cochrane Collaboration, Copenhagen, Denmark), which has proper standardized quality and risk of bias table.

SYRCLE is composed of 10 main items and utilizes “yes” (Y) for a low risk of bias, “no” (N) for a high risk of bias or “unclear” (U) for non-sufficient details to measure the risk of bias. CAMARADES is not a tool with fixed items and may contain different evaluation criteria according to the selected studies. We used nine items that include sampling, blinding and protocol suitability methods according to the outcome of interest, a "present" (1) or "absent" (0) scoring system was used for each evaluation criterion and an arithmetic sum is made at the end. RevMan considers 7 items and uses “H” for a high risk of bias, “L” for a low risk of bias or “U” for an unclear risk of bias, classifying with colors (red, green or yellow, respectively) each item.

### Statistical analyses

All analyses were conducted with Revman Software, version 5.3, and Comprehensive Meta-Analysis Software (Biostat, Englewood, USA), version 3. We calculated the mean differences (MD) with 95% confidence interval (CI), and reported the p-value for the comparison between groups.

We used random effects model to estimate the pooled-effects due to significant heterogeneity between models of DOX-induced cardiotoxicity and exercise protocols. Between-study heterogeneity was explored by Chi-square test and quantified with inconsistency measure (I^2^). I^2^ values of 25%, 50%, and 75% indicate low, moderate, and high heterogeneity, respectively^32^.

Sensitivity analyses were performed to identify outlier studies according to the mean difference of FS between control and intervention groups, and the potential independent predictors of exercise training cardioprotective effects in DOX-treated animals. Thus, we compared the mean difference on FS within subgroups according to the features of animal model (gender and cumulative dose of DOX) and exercise training protocol (modality, duration and timing of execution); the moment of final cardiac function assessment (in relation to the last dose of DOX the animals have received) and the quality scores of the studies according to CAMARADES. Next, we conducted subgroup analyses to confirm the robustness of these findings. The publication bias was assessed following Egger’s regression analysis and identification of funnel plot symmetry.

Finally, we performed a pooled difference power analysis for future pre-clinical studies of DOX-induced cardiomyopathy with WinPepi software, version 11.65^33^. Statistical significance was set at p < 0.05 for all analyses.

## Results

### Search and study selection processes

Fourteen publications^16–29^ were included in quality overview. Of these, 13 studies^16–23,25–29^ could be included in the quantitative synthesis (meta-analysis), since it was not possible to contact the authors nor retrieving the FS data from the graphs from one study^24^ (Fig. 1).

### Description of the included studies

A summary of the characteristics of the 14 included studies is shown in Table 1. The publication year ranged from 2008 to Jan, 2021. In total, the 14 included studies used 923 rodents; This meta-analysis included 835 rats (89.7%) and 88 mice (9.4%), a population of 923 rodents. Of the studies including rats, the majority used Sprague–Dawley (n = 769, 92.1%)^17–25,27^, while the most frequent mice lineage used was C57BL/6 (n = 72, 81.8%)^16,28,29^.

**Table 1:** Summary of the main characteristics of the included studies.

References	Species, gender	Age or Weight	DOX Cumulative Dose, route	Timing	Modality	Duration in weeks (sessions)	Training Protocol	Final ECO
Dolinsky et al., 2013^17^	Mice (C57BL/6), F	10 weeks	32 mg/kg, IP	During and after DOX	AET—Treadmill	8 (40)	Acclimation: 5 days at 10 m/min for 30 min/day. Progressive treadmill during the morning to 18 m/min at 0% grade for 45 min 5 days/week	1 day
Hallet al., 2019^18^	Rats (Sprague–Dawley), F	175–200 g	15 mg/kg, IP	Before DOX	AET—Wheel	16	Acclimation: 1 week. Free access, voluntary, 24 h/day	5 days
Hayard et al., 2011^19^	Rats (Sprague–Dawley), M	25 days	14 mg/kg, IP	During DOX	AET—Wheel	10	Free access, voluntary, 24 h/day	1 day
Hydock et al., 2008^20^	Rats (Sprague–Dawley), M	250–350 g	10 mg/kg, IP	Before DOX	AET—Treadmill or Wheel	10 (50)	Acclimation: 5 days at 13 m/min for 10–15 min. TM: progressive treadmill to 20–30 m/min, 20–60 min, 0–18º grade for 5 days/week. WR: housed 1 per cage with free access to voluntary wheel 24 h/day	5 to 10 days
Hydock et al., 2011^21^	Rats (Sprague–Dawley), M	250 g	10 mg/kg, IP	Before DOX	AET—Treadmill or Wheel	10 (50)	TM: progressive (20–30 m/min to 20–60 min) at 0-18º grade for 5 days/week WR: Free access, voluntary, 24 h/day	4 weeks
Hydock et al., 2012^22^	Rats (Sprague–Dawley), F	200 g	15 or 20 mg/kg, IP	During DOX	AET—Wheel	10	Free access, voluntary, 24 h/day	10 weeks
Jensen et al., 2013^23^	Rats (Sprague–Dawley), F	10–11 weeks	10 mg/kg, IP	Before DOX	AET—Treadmill or Wheel	10 (50)	TM: 5 days/week at 13 m/min up to a 5% grade for 20 min/day gradually increasing throughout weeks 1–4 and 30 m/min up to a 18% grade for 60 min during weeks 5–10. WR: Free access, voluntary, 24 h/day	1 to 9 days
Lien et al., 2015^24^	Rats (Sprague–Dawley), M	10 weeks	10 or 15 mg/kg, IP	Before DOX	AET—Treadmill or Wheel	0.71 (5)	TM: progressive during the dark cycle to 20–60 min/day for 5 days, 18–24 m/min, 0% grade. WR: Free access, voluntary, 24 h/day	5 days
Matsuura et al., 2010^25^	Rats (Sprague–Dawley), M	12 weeks	10 mg/kg, IP	After DOX	AET—Treadmill	6 (30)	Progressive with velocity increased 50% to 60% of maximal velocity with 0% grade for 5 days/week, 60 min/day	-
Morton et al., 2018^26^	Rats (Sprague–Dawley), F	6 months	20 mg/kg, IP	Before DOX	AET—Treadmill	1.42 (10)	Acclimation: 5 consecutive days (10, 20, 30, 40 and 50 min/day on days 1–5, respectively). Two days of rest. 60 min/day at 30 m/min, 0% grade (estimated work rate of 70% VO_2_máx) with two days of rest after day 5 of training	2 days
Parry et al., 2015^27^	Rats (Fischer 344), F	11 weeks	12 mg/kg, IP	Before DOX	AET—Wheel	11	Free access, voluntary, 24 h/day, 7 days/week	1 to 5 days
Pfannenstiel et al., 2018^28^	Rats (Sprague–Dawley), M	10 weeks	12.5 mg/kg, IP	Before DOX	Elevation of food and water	12	Cages allowing for a progressive elevation (from 20.32 to 28 cm) of food and water. Cage height remained at 28 cm for one week. Next, the height was raised by 2.5 cm, and incremental 2.5 cm occurred every 3 days until 35.5 cm, which continued for additional 10 weeks	5 days
Sturgeon et al., 2014^29^	Mice (C57BL/6), M	6–8 weeks	4 mg/kg, IP	During DOX	AET—Treadmill	2 (10)	10 m/min for 45 min, 5 days/week	2 days
Wang et al., 2018^30^	Mice (C57BL/6 J, U; Athymic Swiss Nude, M),	4 weeks	10 mg/kg, IP	During DOX	AET—Treadmill	2 (10)	12 m/min, 0% grade, 5 days/week with 2 consecutive days of rest	1 day

*F* female, *M* male, *U* unclear, *IP* intraperitoneal, *DOX* doxorubicin, *AET* aerobic training, *TM* treadmill, *WR* wheel running, *FS* fractional shortening, *ECO* echocardiography.

With regard to the experimental groups, 469 animals (50.8%) were allocated into the sedentary group and 454 (49.2%) to the trained group. Of the studies involving rats, five^17,21,22,25,26^ used females (n = 379, 45.4%) and six^18–20,23,24,27^ used male animals (n = 456, 54.6%). Of the studies involving mice, only one^16^ used females (n = 22, 25%), and two^28,29^ used male (n = 50, 56.8%) animals. However, we could not retrieve this information from all included studies, as Wang et al.^29^ was lacking this information. Consequently, 16 mice (18.2%) were not classified by gender^29^. Age was mentioned in 10 studies^16,18,22–29^, which ranged from 25 days to 6 months old. Finally, baseline body weight was described in 4 studies^17,19–21^, ranging from 175 to 350 g.

The cardiomyopathy animal model followed the intraperitoneal administration of DOX protocol in all included studies. The cumulative dose of DOX ranged from 4 to 32 mg/kg (mean 12.1 mg/kg). However, one study^28^ used less than 10 mg/kg, which was the modal value of the cumulative doses. The protocols for anthracycline-induced cardiomyopathy were highly heterogeneous regarding the fractioning of the cumulative dose, frequency of injections and timing of administration in relation to the exercise protocol. DOX administration was reported to occur prior^16,24^, concomitantly^18,21,28,29^, and after the end of training protocol^17,19,20,22,23,25,27^. The precise moment when the last cardiac function assessment in relation to the last DOX injection has occurred (in hours or days) was not accurately described among the included studies. Yet, all studies had mentioned if it has occurred after the end of exercise training protocol, after DOX protocol and/or before euthanasia.

Regarding exercise modalities, most of the studies (91.7%) included aerobic exercise training as follows: supervised treadmill running (33.3%)^16,25,28,29^, voluntary wheel running (33.3%)^17,18,21,26^ or supervised treadmill running and voluntary wheel running (33.3%)^19,20,22,23^. Only one study reported to use strength training^27^. The velocity of supervised treadmill running with a progressive transition was mentioned in 8 studies^16,19,20,22,23,25,28,29^, which ranged from 10 to 30 m/min. The inclination of supervised treadmill was reported in 7 studies^16,19,20,22,23,25,29^, and variated from 0 to 18º. The duration of each exercise session was described in 8 studies^16,19,20,22,23,25,28,29^, and it was mostly performed at 5 days/week and lasted between 20 to 60 min/session. The duration of the complete exercise protocol ranged from five days to four months, as follows: 5 days^23^, 10 days^25^, 2 weeks^28,29^, 8 weeks^16^, 10 weeks^18–22^, 11 weeks^26^, 12 weeks^27^, 16 weeks^17^. Three studies with supervised treadmill had reported to include a period of 5 days for habituation to training^16,19,25^, and only one study mentioned the same habituation protocol to supervised treadmill and voluntary wheel running trained animals^19^. Although none of the studies involving voluntary wheel running reported a period or protocol of acclimatization, all of them had referred free voluntary access 24 h/day. Non-exercised (sedentary) animals were used as the controls in all studies.

### Assessment of study quality

In order to minimize poor reporting problems in preclinical studies and to classify most of the items of the risk of bias tools as “unclear”, we decided to use three different tools, SYRCLE, RevMan and CAMARADES. SYRCLE risk of bias regarding randomization and allocation concealment were inadequate to judge for all studies, which implied in unclear risk. Baseline characteristics were considered equal between control and intervention groups, and consequently assessed with low risk of bias. Two studies reported to have animal cages not randomly placed^22,27^. Most of the studies did not reported blinding strategies for caregivers and/or investigators regarding the intervention each animal received, and only one study^16^ provided information on blinding of the examiner of the main outcome. Most of the studies reported to not have conflict of interest regarding protocols and funders. However, for those studies which did not have an explicit conflict of interest statement^19,21,24^ the risk of bias was considered unclear (Supplementary Table 2).

**Figure 2:**
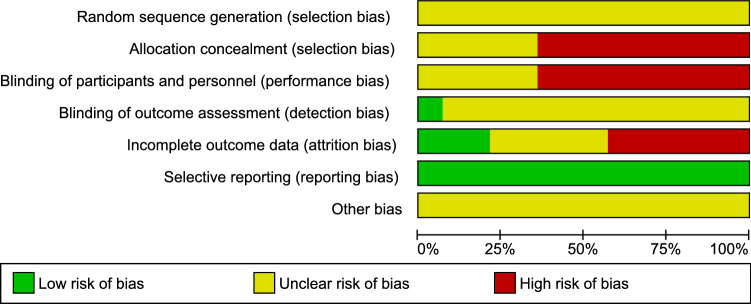
Study quality graph based on Cochrane Collaboration criteria.

According to RevMan quality assessment (Fig. 2), the included studies were classified as having unclear or high risk of bias according to allocation concealment or blinding of participants and personnel. Only one study^16^ was classified with low risk of bias as the authors clearly described blinding strategy for the assessment of outcome, and all studies presented low risk of bias regarding selective reporting. Finally, random sequence generation and presence of other bias remained unclear for all studies as they all lack this information.

Regarding CAMARADES, four studies^19,21,23,24^ were underscored, mostly due to the absence of description of blinded assessment of outcomes, monitoring of body weight parameters and sample size calculation. None of the studies received a maximum score, but two^16,29^ were rated with 8 points, as only sample size calculation was not provided (Supplementary Table 3).

### Efficacy of exercise training on the cardiac function of preclinical models of DOX-induced cardiomyopathy

**Figure 3:**
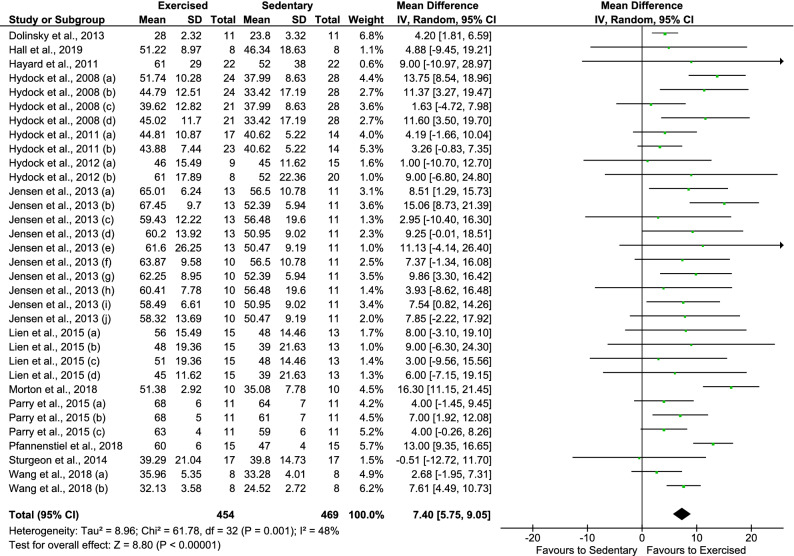
Overall effects of exercise training in fractional shortening of cardiac function of experimental models of doxorubicin-induced cardiomyopathy. Hydock et al., 2008 treadmill group (**a**) at day 5 and (**b**) at day 10, (**c**) wheel running group at day 5 and (**d**) at day 10; Hydock et al., 2011 (**a**) treadmill group, (**b**) wheel running group; Hydock et al., 2012 (**a**) 15 mg/kg and (**b**) 20 mg/kg cumulative dose of DOX; Jensen et al., 2013 treadmill group (**a**) at day 1, (**b**) at day 3, (**c**) at day 5, (**d**) at day 7 and (**e**) at day 9, wheel running group (**f**) at day 1, (**g**) at day 3, (**h**) at day 5, (**i**) at day 7 and (**j**) at day 9; Lien et al., 2015 treadmill group receiving (**a**) 10 mg/kg and (**b**) 15 mg/kg cumulative dose of DOX, wheel running group receiving (**c**) 10 mg/kg and (**d**) 15 mg/kg cumulative dose of DOX; Parry et al., 2015 wheel running group (**a**) at day 1, (**b**) at day 3 and (**c**) at day 5; Wang et al., 2018 mice (**a**) C57BL/6 and (**b**) Athymic Swiss Nude.

Thirteen studies^16–23,25–29^ investigating the effect of exercise training on FS in animals with DOX-induced cardiomyopathy were included in the meta-analysis. Overall, pooled analysis of individual effect sizes of all exercise training protocols showed that this intervention favors DOX-treated animals with regard to reduction of FS in comparison to sedentary state (n = 923, MD = 7.40%, 95% CI 5.75–9.05, p < 0.001) (Fig. 3). The heterogeneity between the studies was considered moderate (I^2^ = 48%)^32^, reflecting the possible anticipated differences between training strategies, timing of outcome assessment and features of animal model.

**Figure 4:**
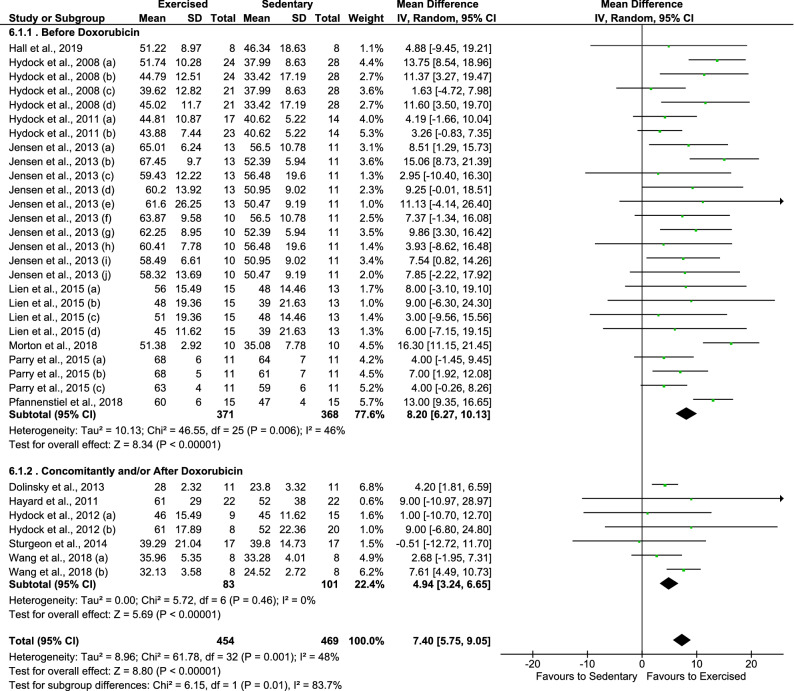
Effects of the timing of exercise protocol in relation to doxorubicin exposure (before vs. concomitantly and/or after) on fractional shortening of animals with doxorubicin-induced cardiomyopathy.

Importantly, exercise training has greater efficacy (test for subgroup differences, p = 0.010) on attenuating DOX-induced FS impairment if executed before DOX exposure (MD = 8.20%, 95% CI 6.27–10.13), when compared to concomitantly and/or after DOX treatment (MD = 4.94%, 95% CI 3.24–6.65)—Fig. 4.

**Figure 5:**
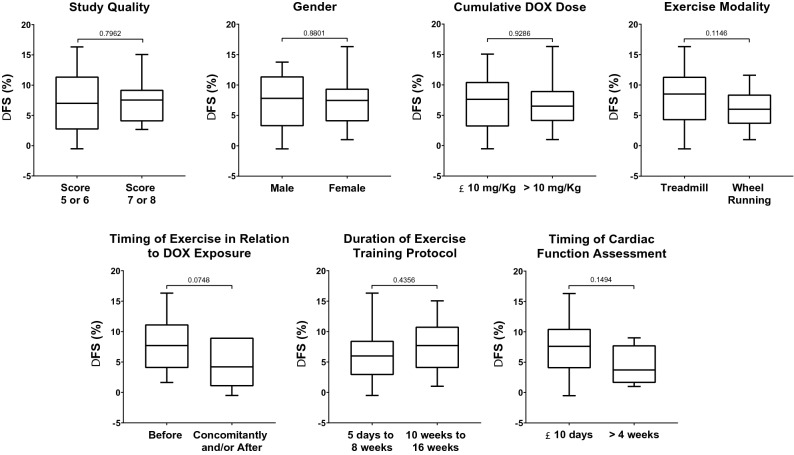
Sensitivity analysis of changes in fractional shortening (FS) based on clinical predictors of exercise training success. Unpaired Student’s T test, p < 0.05.

Subsequently, in order to identify the possible factors which might have influenced on exercise-mediated preservation/improvement of cardiac function following DOX treatment, we conducted an exploratory sensitivity analysis (Fig. 5). We retrieved the mean difference in FS (%) between sedentary and exercised DOX-treated animals for each variable of interest. The timing when exercise was performed in relation to DOX exposure (before *vs.* concomitantly and/or after; p = 0.010) was identified as the only possible factor influencing on exercise-mediated protective efficacy on cardiac function in DOX-treated animals.

Regarding subgroup analyses, modality of the exercise (supervised treadmill or voluntary wheel running) with overall MD = 7.03%, 95% CI 5.45–8.61, p = 0.060 (Table 2) and total duration of the exercise program (overall MD = 7.40%, 95% CI 5.82–9.58, p = 0.610) had no influence on the main outcome (Table 2). Details can be seen in supplementary material (Figures S1 and S2). Also, the features of the animal model are decisive to the results interpretation and extrapolation of these data to clinical landscape. We were able to subgroup the studies according to four animal model-related variables: animal species, gender, cumulative DOX dose and timing of cardiac function assessment. Of those, exercise training exerted cardioprotective effects irrespective to gender (overall MD = 7.64%, 95% CI 5.97–9.32, p = 0.980) (Table 2), and cumulative DOX dose (overall MD = 7.40%, 95% CI 5.75–9.05, p = 0.910) (Table 2). Conversely, the timing of final cardiac function was associated with a greater effect size regarding the preservation of cardiac function when considering the studies which had used rats or that had the echocardiography analysis performed up to 10 days after the completion of all protocols (MD = 7.89%, 95% CI 6.11–9.67, p = 0.020)—Table 2. Details can be seen in supplementary material (Figures S3, S4 and S5).

**Table 2:** Summary of the subgroup analyses on fractional shortening from the included studies.

	*n* studies	Animals exercised/sedentary	Random IV, CI 95% effects	Test overall effects Z (*p*)	Heterogeneity			
					Tau^2^	Chi^2^	*p*	*I*^2^
**Modality of training protocol, MD**								
Treadmill	15	214 / 205	8.42 [5.71, 11.13]	6.10 (p < 0.001)	14.50	38.30	0.0005	63%
Wheel Running	17	225 / 249	5.34 [3.60, 7.07]	6.04 (p < 0.001)	0.00	9.36	0.900	0%
**Duration of exercise protocol, MD**								
5 days – 8 weeks	9	114 / 106	6.68 [3.24, 10.11]	3.81 (p = 0.001)	13.15	22.09	0.005	64%
10 weeks – 16 weeks	24	340 / 363	7.70 [5.82, 9.58]	8.04 (p < 0.001)	7.41	37.37	0.030	38%
**Animal gender, MD**								
Male Only	14	352 / 254	7.64 [4.99, 10.30]	5.64 (p < 0.001)	10.63	26.63	0.010	51%
Female Only	18	194 / 207	7.61 [5.39, 9.83]	6.71 (p < 0.001)	8.24	30.15	0.030	44%
**Cumulative dose of doxorubicin, MD**								
Doxorubicin ≤ 10 mg/Kg	21	308 / 309	7.28 [5.41, 9.16]	7.61 (p < 0.001)	5.39	29.26	0.080	32%
Doxorubicin > 10 mg/Kg	12	146 / 160	7.50 [4.27, 10.74]	4.55 (p < 0.001)	16.20	32.52	0.0006	66%
**Timing of final cardiac function assessment by echocardiography, MD**								
≤ 10 days	29	397 / 406	7.89 [6.11, 9.67]	8.68 (p < 0.001)	9.51	55.85	0.001	50%
> 4 weeks	4	57 / 63	3.60 [0.44, 6.75]	2.23 (p = 0.030)	0.00	0.70	0.870	48%
**Study quality**								
Score 5 or 6	14	234 / 254	7.46 [4.13, 10.80]	4.39 (p < 0.001)	20.09	30.65	0.004	58%
Score 7 or 8	19	220 / 215	7.21 [5.75, 9.05]	7.72 (p < 0.001)	5.52	30.50	0.030	41%

*MD* mean difference, *IV* inverse variance method, *CI* confidence interval.

### Effects of study quality

We also investigated the influence of the study quality (according to the items on the CAMARADES checklist) on the main outcome (Table 2 and Figure S6, supplementary material), and we identified that the cardioprotective effects of exercise occurred independently on the study quality score (MD = 7.40%, 95% CI 5.75–9.05, p = 0.900).

### Mechanisms of exercise-mediated cardioprotection in rodents with DOX-induced cardiomyopathy

A summary of the main findings and possible mechanisms implicated on exercise-mediated cardioprotection reported by each included study are presented in Supplementary Table 4. All studies described the exercise-mediated systemic, functional or molecular mechanisms possibly implicated in attenuation, preservation or improvement of FS in DOX-treated animals. Exercise-mediated reduction of DOX-accumulation was the most reported mechanism at cellular level^17,22,25,26,29^, followed by preservation of myosin heavy chain^19–21,27^. Three studies^16,20,23^ evaluated the association between exercise training and calcium homeostasis^20,23^, with a particular focus on the preservation of myocardial contraction and global cardiac function in exercised DOX-treated animals. One study^18^ mentioned that the exercise was not able to protect against DOX-induced impairments on body composition and weight gain. One study^28^ stated that exercise did not reverse the cardiotoxic effects of DOX, although it was associated with upregulation of myocardial protein kinase B activity, which is involved with cell survival.

### Publication bias

Analysis of publication bias was found to be influenced by the presence of small study effects. Symmetric funnel plots indicated the absence of publication bias for FS (Egger’s regression test, 2-tailed, p = 0.675) (Figure S7, supplementary material).

### Power calculation

Based on our results, we performed a sample size calculation for future preclinical studies of exercise training as intervention for DOX-induced cardiotoxicity. To obtain a power of at least 90% in a two-sided two-sample t-test with an alpha of 0.05, 9 and 11 animals needed to be included in each group to detect a significant mean difference in FS of 8.08% in rats and 4.83% in mice, respectively.

### Clinical trials

Supplementary Table S5 summarizes all registered clinical trials (http://clinicaltrials.gov/) which aim to evaluate the role of exercise training in cardiac function in oncological patients undergoing anthracyclines-based regimens. For this search, we used the terms “cardiotoxicity” and “exercise” (including the term “physical activity”), which returned 23 registered studies between 2008 and 2020. Of these, only 15 studies have been designed to evaluate exercise in an interventional fashion, i.e., investigating the preventive/prophylactic effects of this practice against the possible harmful effects of antineoplastic agents on cardiovascular function. To date, only 3 of these studies have been completed, and however, none of these has published results. Furthermore, these studies are significantly distinct regarding the employed exercise protocols, which means that the chosen modalities, duration, intensity and adherence to program are highly heterogeneous. It highlights the existence of an unmet need for a high quality body of evidence to support the decision-making about the ideal training protocol for cancer patients, including the definition to what extent exercise prescription may protect the cardiac function of patients with a history of sedentary behavior.

## Discussion

To the best of our knowledge, this is the first study to systematically review the effects of physical exercise on the cardiac function of preclinical models of DOX-induced cardiomyopathy. After a comprehensive search and data assimilation from 15 studies, the main findings were: (1) DOX-treated animals who have undergone exercise training presented a 7.40% mean difference in FS in comparison to sedentary DOX-treated animals; (2) subgroup analyses of the effects of exercise features suggested a greater cardioprotective effect of exercise training prior to DOX exposure (*vs.* concomitantly and/or after); (3) subgroup analyses of the timing of final cardiac function assessment suggested that a more pronounced effect size of exercise training on FS is likely to occur in those studies which echocardiography is performed up to 10 days after all experimental protocols completion (i.e., DOX exposure and exercise training protocol).

A larger effect size of exercise-mediated cardioprotection against DOX was achieved in those groups who trained before DOX exposure and followed a programed, scheduled and supervised modality of exercise. Preconditioning resulted in a mean difference almost twice greater in FS when compared to groups who trained concomitantly and/or after DOX treatment. The generation of massive amounts of reactive oxygen species (ROS)^34,35^, disruption of calcium transport in the sarcoplasmic reticulum^20^ and mitochondrial energy metabolism^25^ are well known features of anthracyclines-induced cardiotoxicity. On its turn, exercise may decrease DOX accumulation in cardiac tissue, and therefore protect against ROS generation, while stimulates antioxidants production^36^ and increases the resistance to ROS-induced apoptotic stimuli due to metabolic adaptations^37^. Therefore, regular exercising before DOX exposure may have a role in cardiomyocytes’ conditioning to DOX insults, which may have resulted in a superior response against the deleterious effects of this anthracycline, and ultimately in the preservation of cardiac function.

Another key find of this meta-analysis refers to the superiority of programmed and supervised aerobic training in relation to the voluntary modality. Per definition, while physical activity is any bodily movement produced by skeletal muscles with energy expenditure requirement, physical exercise refers to a planned, structured, repetitive subcategory of physical activity^38^. Yet, current evidence supports that both physical activity and exercise harbor a plethora of benefits for both healthy individuals and patients compared to a sedentary life^39^. Nevertheless, among individuals who exercise, those who exercise under supervision are more likely to achieve better results in terms of aerobic capacity, muscle strength, functional activity, loss of fat mass, health related quality of life and pain^39–43^.

Regarding experimental studies, it is possible to consider voluntary wheel running as physical activity, and supervised treadmill running as exercise. Supervised treadmill running is a strategy that implies great stimulus and intensity of effort, and most of the experimental studies investigating this modality of exercise aim for moderate intensity^44^. These protocols are more complex than those on free wheel running, since a period of adaptation is required and the intensity is not defined by the animal. In counterpart, voluntary wheel running is a low intensity aerobic training modality. Collectively, these results suggest that future clinical trials must be designed to include regular, planned and supervised exercise training protocols prior to DOX-based regimens in order to acquire superior cardiac adaptation and enhancement of cardioprotective defenses.

Conversely to expected, exercise duration was not identified as an independent predictor of cardiac outcome. The stratification of the studies into subgroups was very challenging. First, because the data on the number of sessions and the duration of each session were only possible to retrieve in those studies with forced exercise modalities. Still, the protocols were highly heterogeneous, an so it was the duration of each protocol, which occurred for 5 days^23^, 10 days^25^, 2 weeks^28,29^, 8 weeks^16^ and 10 weeks^19,20,22^. Second, because the studies which has investigated the effects of exercise using a voluntary wheel running did not present enough details to provide a complete depiction of the training protocol. Also, voluntary wheel running protocols did not specify the average time that each animal remained active on the wheel, nor did they inform whether each cage contained a single animal to avoid competition and the ideal control of distance traveled and frequency. Longest training periods (i.e., 16 weeks^17^ and 10 weeks^18–22^) occurred in studies of low intensity voluntary wheel running. As even at low intensity exercise can induce favorable metabolic adaptations^45^, it is likely that the similar effect size exerted by exercise on cardiac function denotes an equivalence of cumulative cardioprotective effects between longer periods of low intensity and shorter periods of moderate intensity exercises^46^.

All included studies had investigated molecular and cellular mechanisms possibly implicated in exercise-mediated cardioprotection, including reduction of DOX in myocardial tissue accumulation^17,22,25,26,29^, maintenance of heavy chain myosin^19–21,27^ and calcium homeostasis integrity^16,20,23^, modulation of oxidative stress response^36^ and resistance to apoptotic stimuli induced by reactive oxygen species (ROS) due to metabolic adaptations^37^. Overall, is it likely that cumulative exercise benefits might preserve the contractile capacity and overall cell integrity, since the lipoperoxidation triggering and hydrogen peroxide production would occur at tolerable levels^46^ (See Supplementary Table 4 for main results and possible mechanisms implicated on exercise-mediated influence on cardiac function).

We also explored the influence of the features of animal model on cardiac function of DOX-treated trained animals, i.e., species, sex, cumulative dose of DOX and timing of final cardiac function assessment. Although the role of estrogen on gender-related cardioprotection^47^, including in the context of DOX-induced cardiomyopathy, is well documented^48^, FS was similar between male and female animals. None of the studies using female animals^16,17,21,22,25,26^ discussed the choice of this gender, took into consideration the influence of female hormones in the outcomes of interest or reported particular methodological/monitoring aspects, such as handling estrous cycle. The consideration of female animals in cardiotoxicity pre-clinical studies is of high relevance since anthracyclines are the backbone of chemotherapy-based regimens for breast and ovarian neoplasms^49^.

The experimental protocol for DOX-induced cardiomyopathy employed different fractioning and cumulative doses. The average body area of an adult cancer patient is approximately 1.72 m^2^ (average weight 66.3 kg)^50^, and that DOX treatment regimens do not usually exceed 550 mg/m^2^ or 14.26 mg/kg^51^. Although 5 studies have used cumulative doses higher than which is predicted for humans (15–32 mg/kg)^16,17,21,23,25^, the authors described similar benefits and size effects from exercise training on cardiac function to those studies which have employed lower cumulative doses of DOX. Also, subgroup analysis did not show difference on FS between trained animals exposed to a cumulative dose ≤ 10 mg/kg or > 10 mg/kg of DOX.

The timing of cardiac function assessment in preclinical studies is crucial to define the onset of cardiotoxicity (early or late)^52^ and to accurately reproduce a specific form of disease presentation. Considering the clinical definition of acute cardiotoxicity if it occurs within two weeks, and chronic, if it occurs within one year following the onset of treatment with antineoplastics^3^, most of the included studies in this meta-analysis have reproduced the early onset of cardiotoxicity. Subgroup analysis revealed that the mean difference on FS between groups was greater in those studies which had evaluated the cardiac function up to 10 days after all experiments (i.e., DOX exposure and exercise protocol completion). The long-term assessment of cardiac function in anthracycline-treated patients detects more severe impairment than earlier^53^. Thus, the evaluation of cardiac function up to 10 days after the end of experimental protocols might reflect the incomplete remodeling/adaptation process rather than the actual exercise cardioprotective effects against DOX. In this regard, several issues remained to be elucidated: (1) the long-term effects of exercise on DOX-induced cardiac function impairments, as short-term echocardiographic assessments may have overestimated cardiac adaptability; (2) the effects of detraining, particularly in those protocols of preventive exercise which are suspended during DOX exposure^54^.

Left ventricle function of anthracyclines-treated patients is routinely monitored by echocardiogram or multigated acquisition scan measures (MUGA)^55^. Despite the decrease in left ventricular (LV) ejection fraction (LVEF) being the main parameter used to diagnose and classify cardiotoxicity in humans, only two studies^16,29^ in our selection reported this measure to represent changes in cardiac function. Thus, FS was the most reported result to quantify the global function of the left ventricle in animal models of DOX-induced cardiomyopathy. FS reflects LVEF when the left ventricle has homogeneous contraction and, as a global analysis, represents any diastolic dimension lost in systole^56,57^.

Although most of the included studies affirm that exercise may have a role in DOX-induced cardiotoxicity prevention and management, only six^19,22,25–27,29^ showed significant differences in mean FS between sedentary and exercised animals treated with DOX. It might implicate that exercise-mediated cardioprotective effects were mostly supported by other experimental results (isolated working hearts, Doppler flow images and mechanistic investigations at tissue and cell levels) rather than exclusively by the in vivo assessment of cardiac function. As the use of FS in the clinical practice is limited due to poor representation of global shortening of the LV in the presence of abnormalities of the wall and overestimation of general LV function^58^, further experimental studies must consider to standardize the assessment of cardiac function with greatest similarity as possible to clinical scenario.

Generally, higher study quality yields, more efficient evaluation on preclinical study effects and lower quality study normally overestimates intervention or experimental effects^59,60^. However, subgroup analysis comparing studies with low (5–6) and high (7–8) CAMARADES scores, no differences were observed in cardioprotection effect according to quality score. Even in those studies with high CAMARADES quality scores, publication bias analysis showed a disparity between studies, which reflect individual variation between studies (features of animal model, exercise protocols, etc.).

According to *clinicaltrials.gov*, there are 13 ongoing studies investigating exercise training for patients under treatment with anthracyclines. The vast majority includes patients with breast cancer, which is a population of older individuals with high incidence of cardiovascular risk factors^61,62^. However, the exercise protocols are highly heterogeneous among these studies in terms of modality, intensity, duration and timing of execution in relation to chemotherapy.

Future preclinical and clinical studies must focus on the influence of sex hormones and the presence of comorbidities in the susceptibility, onset and severity of cardiotoxicity^63^, as most of cardiac and cancer risk factors overlap and interfere in both cardiovascular and oncological outcomes^64^. However, due to lack of information on the independent impact of each risk factor within the landscape of preventive/therapeutic exercise for DOX-induced cardiovascular injuries, future preclinical studies must include comorbidities and other risk factors to enable clinical translation of the results.

Also, although clinical evidence is heterogeneous, exercise has been recommended to patients with a cancer diagnosis. In this regard, it is still unclear if for sedentary patients undergoing anthracyclines-based regimens if early exercise-mediated myocardial and vascular adaptations may act as a double-edged sword and potentialize the deleterious effects of anthracyclines.

Even though our meta-analysis adds relevant results to the current state-of-art, we are aware of its limitations. First, it is essential to highlight that as other meta-analysis of preclinical studies, studies with experimental animals are most likely to be published if they present positive results. Thus, it may have contributed as a major source of bias. Second, the confirmation of cardiotoxicity was based on the echocardiographic measurement of FS, which is a highly sensitive and observer-dependent method, and might have contributed to the inter-studies heterogeneity (48%). In fact, LVEF rather than FS is the gold-standard measurement of cardiac function which is assessed for cardiotoxicity diagnosis in the clinical setting. Also, none of the studies provided a definition of cardiotoxicity based on a cutoff value for FS (e.g., drop from baseline or < than a specific value) and/or included a classic biomarker of cardiac damage (cardiac troponins, BNP). Finally, almost all studies included studies included only healthy and young animals, i.e., no study had included tumor-bearing animals or one of the extensively described shared risk factors of cancer and cardiovascular diseases, such as older age, comorbidities (diabetes, obesity, hypertension, etc.) and low levels of female hormones.

In conclusion, this meta-analysis presented evidence on the cardioprotective effects of exercise in animal models of DOX-induced cardiotoxicity, particularly due to preservation of global cardiac function. Exercise-mediated cardioprotection against deleterious effects of DOX on myocardium were irrespective of total duration of the aerobic exercise protocols. In counterpart, despite performed for shorter periods, planned moderate intensity aerobic exercise exerted superior cardioprotective effects in DOX-treated animals in comparison to low intensity, voluntary/unsupervised exercise training performed for longer periods. Further studies still have to improve the control over possible sources of heterogeneity, including standardization of protocols for cardiotoxicity induction and incorporation of clinical definition criteria in preclinical models (assessment of LVEF and biomarkers of cardiac damage). Also, in order to accelerate clinical translation of preclinical results, these studies must include tumor-bearing animals and animal models reproducing the clinical features of most cancer patients (older age, menopause, comorbidities, etc.).

## Supplementary Information

Supplementary Information
