# Supplementary material for: Cardioprotective effects of exercise training on doxorubicin-induced cardiomyopathy: a systematic review with meta-analysis of preclinical studies
Source: Sci Rep. 2021 Mar 18;11:6330. doi: 10.1038/s41598-021-83877-8 (PMC7973566; doi:10.1038/s41598-021-83877-8)
Supplement: Supplementary file 1 — Supplementary Information [file 41598_2021_83877_MOESM1_ESM.pdf]

Cardioprotective effects of exercise training on doxorubicin-induced cardiomyopathy: a systematic review with meta-analysis of preclinical studies

Paola Victória da Costa Ghignatti [1]

Laura Jesuíno Nogueira [1]

Alexandre Machado Lehnen [1]

Natalia Motta Leguisamo* [1]

[1] Post-Graduate Program in Health Sciences: Cardiology, Institute of Cardiology of Rio Grande do Sul/University Foundation of Cardiology, Porto Alegre, Rio Grande do Sul, Brazil.

***Correspondent Author**

Dr Natalia Motta Leguisamo
Institute of Cardiology of Rio Grande do Sul/University Foundation of Cardiology.
Av. Princesa Isabel, 370, Porto Alegre, Rio Grande do Sul, Brazil. CEP 90620-001.
E-mail: nmleguisamo@gmail.com

Supplementary Table 1 – Detailed search strategy

PubMed

"("Doxorubicin"[mesh] OR (Farmiblastina) OR (Ribodoxo) OR (Rubex) OR (Adriamycin) OR (Adriblastin) OR (Adriblastine) OR (Adriblastina) OR (Adriablastine) OR (Adriablastin) OR (Adrimedac) OR (DOXO-cell) OR (DOXO cell) OR (Urokit Doxo-cell) OR (Urokit Doxo cell) OR (Doxolem) OR (Doxorubicin Hexal) OR (Doxorubicin Hydrochloride) OR (Hydrochloride, Doxorubicin) OR (Doxorubicin NC) OR (Doxorubicina Ferrer Farm) OR (Doxorubicina Funk) OR (Doxorubicina Tedec) OR (Doxorubicine Baxter) OR (Doxotec) OR (Myocet) OR (Onkodox)) and ("Exercise"[mesh] OR (Exercises) OR (Physical Activity) OR (Activities, Physical) OR (Activity, Physical) OR (Physical Activities) OR (Exercise, Physical) OR (Exercises, Physical) OR (Physical Exercise) OR (Physical Exercises) OR (Acute Exercise) OR (Acute Exercises) OR (Exercise, Acute) OR (Exercises, Acute) OR (Exercise, Isometric) OR (Exercises, Isometric) OR (Isometric Exercises) OR (Isometric Exercise) OR (Exercise, Aerobic) OR (Aerobic Exercise) OR (Aerobic Exercises) OR (Exercises, Aerobic) OR (Exercise Training) OR (Exercise Trainings) OR (Training, Exercise) OR (Trainings, Exercise))"

Scopus Database

"TITLE-ABS-KEY (Doxorubicin AND Exercise)"

Web of Science Database

"All fields: (Doxorubicin) *AND* All fields: (Exercise). Overtime: All years."

Supplementary Table 2 – SYRCLE study quality

References	Was the allocation sequence adequately generated and applied?	Were the groups similar at baseline or were they adjusted for confounders in the analysis?	Was the allocation to the different groups adequately concealed during?	Were the animals randomly housed during the experiment?	Were the caregivers and/or investigators blinded from knowledge which intervention each animal received during the experiment?	Were animals selected at random for outcome assessment?	Was the outcome assessor blinded?	Were incomplete outcome data adequately addressed?	Are reports of the study free of selective outcome reporting?	Was the study apparently free of other problems that could result in high risk of bias?
**Dolinsky et al., 2013 [17]**	U	Y	U	U	U	Y	Y	Y	U	U
**Hallet al., 2019 [18]**	U	Y	N	U	N	U	U	Y	U	U
**Hayard et al., 2011 [19]**	U	Y	N	U	N	U	U	N	U	U
**Hydock et al., 2008 [20]**	U	Y	N	U	N	U	U	U	U	U
**Hydock et al., 2011 [21]**	U	Y	N	U	N	U	U	N	U	U
**Hydock et al., 2012 [22]**	U	Y	N	U	N	U	U	U	U	U
**Jensen et al., 2013 [23]**	U	Y	N	N	N	U	U	N	U	U
**Lien et al., 2015 [24]**	U	Y	N	U	N	U	U	U	U	U
**Matsuura et al., 2010 [25]**	U	Y	U	U	U	U	U	N	U	U
**Morton et al., 2018 [26]**	U	Y	U	U	U	U	U	U	U	U
**Parry et al., 2015 [27]**	U	Y	N	U	N	U	U	Y	U	U
**Pfannenstiel et al., 2018 [28]**	U	Y	N	N	N	U	U	U	U	U
**Sturgeon et al., 2014 [29]**	U	Y	U	U	U	U	U	N	U	U
**Wang et al., 2018 [30]**	U	Y	U	U	U	U	U	N	U	U

Y=yes, low risk of bias; U=unclear, unclear risk of bias; N=no, high risk of bias.

Supplementary Table 3 – CAMARADES study quality

References	Peer review publication	Presence of randomization	Assessment of dose-response relationship	Blinded assessment of behavioral outcome	Monitoring of body weight parameters	Sample size calculation	Statement of compliance with regulatory requirements	Statement of potential conflicts of interest	Use of accurate/suitable/adequate dose to animal model	Total
**Dolinsky et al., 2013 [17]**	1	1	1	1	1	0	1	1	1	8
**Hallet al., 2019 [18]**	1	1	1	0	1	0	1	1	1	7
**Hayard et al., 2011 [19]**	1	1	1	0	1	0	1	1	1	7
**Hydock et al., 2008 [20]**	1	1	1	0	0	0	1	0	1	5
**Hydock et al., 2011 [21]**	1	1	1	0	0	0	1	1	1	6
**Hydock et al., 2012 [22]**	1	1	1	0	0	0	1	0	1	5
**Jensen et al., 2013 [23]**	1	1	1	1	0	0	1	1	1	7
**Lien et al., 2015 [24]**	1	1	1	0	0	0	0	1	1	5
**Matsuura et al., 2010 [25]**	1	1	1	0	0	0	1	0	1	5
**Morton et al., 2018 [26]**	1	1	1	0	0	0	1	1	1	6
**Parry et al., 2015 [27]**	1	1	1	0	1	0	1	1	1	7
**Pfannenstiel et al., 2018 [28]**	1	1	1	0	1	0	1	1	1	7
**Sturgeon et al., 2014 [29]**	1	1	1	0	1	0	1	1	0	6
**Wang et al., 2018 [30]**	1	1	1	1	1	0	1	1	1	8

0=no, absence of information, high risk of bias; 1=yes, information present, low risk of bias.

Supplementary Table 4 – Main results and possible mechanisms implicated on exercise-mediated influence on cardiac function

References	Findings
**Dolinsky et al., 2013 [17]**	Exercise increased SERCA2a, 4-Hydroxynonenal and manganese superoxide dismutase.
**Hallet al., 2019 [18]**	Exercise, combined with calorie restriction, increased left ventricular developed pressure, end systolic pressure, and left ventricular maximal rate of pressure development by reduction of DOX accumulation in left ventricle.
**Hayard et al., 2011 [19]**	Exercise preserved cardiac function, however did not protect against the bluting of the overall growth curve or against the bluting of normal organ growth.
**Hydock et al., 2008 [20]**	Exercise attenuated DOX-induced cardiac dysfunction and was associated with a preservation of myosin heavy chain isoform expression.
**Hydock et al., 2011 [21]**	Exercise was cardioprotective against DOX cardiotoxicity due to preservation myosin heavy chain but not sarcoendoplasmic reticulum Ca^2+ ATPase 2a expression.
**Hydock et al., 2012 [22]**	Exercise animals receiving DOX treatments had significantly lower b-myosin heavy chain expression.
**Jensen et al., 2013 [23]**	Cardioprotective effects of exercise against DOX-induced injury may be due, in part, to a reduction in myocardial DOX accumulation.
**Lien et al., 2015 [24]**	Exercise may be a valuable adjuvant therapy to offset acute cardiotoxicities and maintaining calcium handling in cardiomyocytes may be responsible, in part, for the preservation in cardiac function.
**Matsuura et al., 2010 [25]**	Exercise reduced mortality in DOX-induced heart failure animals and restored vascular smooth muscle relaxation properties. However, did not ameliorate intraplatelet nitric oxide bioavailability and endothelial function.
**Morton et al., 2018 [26]**	Exercise protects against DOX-induced myotoxicity and prevent mitochondrial dysfunction. Exercise may induce protection by increasing the expression of mitochondria-specific ATP-binding cassette transporters and reducing mitochondrial DOX accumulation.

Supplementary Table 4 (cont.) – Main results and possible molecular and cellular mechanisms implicated in exercise-mediated influence on cardiac function

References	Findings
**Parry et al., 2015 [27]**	Exercise preserved cardiac function up to 5 days following DOX treatment. Also, reduced ventricular DOX accumulation and upregulated ventricular MPR1 and MPR2. However, did not affect the antitumor efficacy of DOX, it still provided protection against cardiac dysfunction.
**Pfannenstiel et al., 2018 [28]**	Exercise provide protection against DOX-induced cardiac dysfunction, which may be a result of preservation of the cardiac myosin heavy chain isoform distribution.
**Sturgeon et al., 2014 [29]**	Exercise did not reverse the cardiotoxic effects of DOX. However, reduced tuberous sclerosis 2 abundance and increased myocardial protein kinase B activity and the efficacy of DOX in inhibiting tumor growth without mitigating subclinical DOX-induced cardiotoxicity in a murine model of melanoma.
**Wang et al., 2018 [30]**	Exercise mitigates cardiotoxicity, possibly through altered delivery of DOX to myocardial tissue.

Supplementary Table 5 – Registered clinical trials of exercise training for anthracycline-treated patients

Title Acronym	Number Clinical Trial	Population	Exercise Protocol	Status	Notes
-	NCT00728429	All genders; >18-80 years; 4-6 courses of therapy scheduled.	24 weeks program	Terminated	Slow accrual
-	NCT01719562	All genders; >22 years and older; ongoing therapy.	1-2 training sessions/week onsite and 1-2 sessions/week at home (slow 15 min aerobic warm-up followed by 20 min of strength training, 15 min of progressive intensity aerobic exercise and 10 min of cool down)	Recruiting	-
-	NCT02006979	Female; >18 years; Newly diagnosed with stage I-IIIA breast cancer; scheduled to chemotherapy in cycles of 2-3 weeks long.	Acute bout of exercise performed ≤24 hours prior to each cycle of anthracyclines and no exercise for 48 hours post.	Completed	No Study Results Posted Yet
ATOPE	NCT03787966	Female; >18-70 years; I-III stage breast cancer; waiting therapy.	Adapted therapeutic exercise program during cancer treatment (18 bouts of 1'5 hours multimodal components: aerobic, strength and fascial release exercises)	Not yet recruiting	-
EXACT	NCT02471053	All genders; >18-65 years; primary/non-recurrent breast or hematological malignancy; within eight weeks of first anthracycline dose.	Twice a week for 12 weeks (group warm-up activity, followed by 45 min of aerobic activity, moderate intensity, and ending with a cool down)	Completed	No Study Results Posted Yet
EXACT2	NCT03748550	Female; >18 years; non-treated stages I-III breast cancer.	Home-based progressive aerobic exercise program.<br><br>2 times on non-consecutive days a week	Not yet recruiting	-
EXACCT	NCT02842658	All genders; >40-70 years; stages I-III breast cancer; scheduled therapy.	Weekly phone call to stimulate physical activity during chemotherapy; compliance will be verified by diaries and pedometers.	Recruiting	-
HF-PROACTIVE	NCT02796365	All genders; >18-75 years; undergoing or completed the treatment; relative reduction in LV strain >10% or troponin >0.04 ng/mL or an increase of 0.04 ng/mL if baseline troponin is elevated.	3 days per week (interval training on a treadmill or bike at an intensity between 50-90% of heart rate reserve and exercises 1-2 days per week)	Completed	No Study Results Posted Yet

Supplementary Table 5 (cont.) – Registered clinical trials of exercise training for anthracycline-treated patients

Title Acronym	Number Clinical Trial	Population	Exercise Protocol	Status	Notes
CORE	NCT03089502	Women; >18 years of age; stages I-III breast cancer; diagnosis of cardiac toxicity (decrease of left ventricular ejection fraction of >10% to a value < 53%); undergoing known cardiotoxic agents.	Walking or biking program at 60-80% of VO_2 peak, progressing to a minimum of 150 minutes/week. One weekly exercise session will be supervised, and participants will be expected to complete four additional aerobic training sessions at home or in the community each week. Resistance training will be introduced at week 4 (10 exercises targeting all major muscle groups. One weekly resistance training session will be supervised following the aerobic training, and participants will be expected to complete one to two additional sessions at home or in the community each week. Total of 12 weeks.	Terminated	Limited recruitment
-	NCT04036032	All genders; >9 years; treatment completed >2 years ago.	Encouragement to exercise 4-5 times a week for 3 months.	Recruiting	-
ONCORE	NCT03964142	Female; >18-69 years; first stages I-III breast cancer; ongoing treatment.	Cardiac rehabilitation or conventional management with physical exercise recommendation.	Recruiting	-
CAPRICE	NCT03850171	Female; >18 years; breast cancer; ongoing treatment.	Exercise will be performed concurrent to treatment during months 1 to 3, which will consist in 90 min, twice a week (40 min cycling at moderate intensity, increasing on a weekly basis if possible. After, patients continue the exercise session with 40 min of strength training, stretching, relaxation, coordination and balance training. In addition, patients will be instructed to perform at least one additional endurance-related activity per week in their own time with a duration of 30-60 min at moderate intensity levels).	Recruiting	-
-	NCT04047901	All genders; 18 years and older; functional class I-III (NYHA); ejection fraction <55%; received treatment for heart failure after cancer treatment.	16 weeks of training including 40 minutes of aerobic training, 15 minutes of resistive exercise and 5 minutes of relaxation.	Recruiting	-

Supplementary Table 5 (cont.) – Registered clinical trials of exercise training for anthracycline-treated patients

Title Acronym	Number Clinical Trial	Population	Exercise Protocol	Status	Notes
AEROHEMONCO	NCT04476576	All genders; 18 years to 65 years old; new-onset chemotherapy; lymphoma (Hodgkin or non-Hodgkin).	Active group: 3 months, 3 times/week aerobic program. Control group: 3 months, 3 times/week flexibility program.	Not yet recruitment	-
HIMALAYAS-P	NCT04598646	All genders; 18 years to 39 years old; received cancer treatment with known cardiovascular risks in the previous 12 months; be cancer-free at the time of enrollment; ejection fraction <53/54%, global longitudinal strain >–18%, or diastolic dysfunction.	Aerobic exercise programs consisting of two days of supervised, facility-based high-intensity interval training (HIIT) and one day of supervised home-based moderate-intensity continuous training (MICT) per week.	Not yet recruitment	-

Supplementary Figure S1

Study or Subgroup	Exercised Mean	Exercised SD	Exercised Total	Sedentary Mean	Sedentary SD	Sedentary Total	Weight	Mean Difference IV, Random, 95% CI
**5.1.1 . Treadmill**								
Dolinsky et al., 2013	28	2.32	11	23.8	3.32	11	8.1%	4.20 [1.81, 6.59]
Hydock et al., 2008 (a)	51.74	10.28	24	37.99	8.63	28	4.8%	13.75 [8.54, 18.96]
Hydock et al., 2008 (b)	44.79	12.51	24	33.42	17.19	28	2.7%	11.37 [3.27, 19.47]
Hydock et al., 2011 (a)	44.81	10.87	17	40.62	5.22	14	4.2%	4.19 [-1.66, 10.04]
Jensen et al., 2013 (a)	65.01	6.24	13	56.5	10.78	11	3.2%	8.51 [1.29, 15.73]
Jensen et al., 2013 (b)	67.45	9.7	13	52.39	5.94	11	3.8%	15.06 [8.73, 21.39]
Jensen et al., 2013 (c)	59.43	12.22	13	56.48	19.6	11	1.2%	2.95 [-10.40, 16.30]
Jensen et al., 2013 (d)	60.2	13.92	13	50.95	9.02	11	2.2%	9.25 [-0.01, 18.51]
Jensen et al., 2013 (e)	61.6	26.25	13	50.47	9.19	11	1.0%	11.13 [-4.14, 26.40]
Lien et al., 2015 (a)	56	15.49	15	48	14.46	13	1.7%	8.00 [-3.10, 19.10]
Lien et al., 2015 (b)	48	19.36	15	39	21.63	13	1.0%	9.00 [-6.30, 24.30]
Morton et al., 2018	51.38	2.92	10	35.08	7.78	10	4.8%	16.30 [11.15, 21.45]
Sturgeon et al., 2014	39.29	21.04	17	39.8	14.73	17	1.4%	-0.51 [-12.72, 11.70]
Wang et al., 2018 (a)	35.96	5.35	8	33.28	4.01	8	5.3%	2.68 [-1.95, 7.31]
Wang et al., 2018 (b)	32.13	3.58	8	24.52	2.72	8	7.2%	7.61 [4.49, 10.73]
**Subtotal (95% CI)**			**214**			**205**	**52.6%**	**8.42 [5.71, 11.13]**
Heterogeneity: Tau² = 14.50; Chi² = 38.30, df = 14 (P = 0.0005); I² = 63%								
Test for overall effect: Z = 6.10 (P < 0.00001)								
**5.1.2 . Wheel Running**								
Hall et al., 2019	51.22	8.97	8	46.34	18.63	8	1.1%	4.88 [-9.45, 19.21]
Hayard et al., 2011	61	29	22	52	38	22	0.6%	9.00 [-10.97, 28.97]
Hydock et al., 2008 (c)	39.62	12.82	21	37.99	8.63	28	3.8%	1.63 [-4.72, 7.98]
Hydock et al., 2008 (d)	45.02	11.7	21	33.42	17.19	28	2.7%	11.60 [3.50, 19.70]
Hydock et al., 2011 (b)	43.88	7.44	23	40.62	5.22	14	6.0%	3.26 [-0.83, 7.35]
Hydock et al., 2012 (a)	46	15.49	9	45	11.62	15	1.5%	1.00 [-10.70, 12.70]
Hydock et al., 2012 (b)	61	17.89	8	52	22.36	20	0.9%	9.00 [-6.80, 24.80]
Jensen et al., 2013 (f)	63.87	9.58	10	56.5	10.78	11	2.5%	7.37 [-1.34, 16.08]
Jensen et al., 2013 (g)	62.25	8.95	10	52.39	5.94	11	3.6%	9.86 [3.30, 16.42]
Jensen et al., 2013 (h)	60.41	7.78	10	56.48	19.6	11	1.4%	3.93 [-8.62, 16.48]
Jensen et al., 2013 (i)	58.49	6.61	10	50.95	9.02	11	3.5%	7.54 [0.82, 14.26]
Jensen et al., 2013 (j)	58.32	13.69	10	50.47	9.19	11	2.0%	7.85 [-2.22, 17.92]
Lien et al., 2015 (c)	51	19.36	15	48	14.46	13	1.4%	3.00 [-9.56, 15.56]
Lien et al., 2015 (d)	45	11.62	15	39	21.63	13	1.3%	6.00 [-7.15, 19.15]
Parry et al., 2015 (a)	68	6	11	64	7	11	4.5%	4.00 [-1.45, 9.45]
Parry et al., 2015 (b)	68	5	11	61	7	11	4.9%	7.00 [1.92, 12.08]
Parry et al., 2015 (c)	63	4	11	59	6	11	5.8%	4.00 [-0.26, 8.26]
**Subtotal (95% CI)**			**225**			**249**	**47.4%**	**5.34 [3.60, 7.07]**
Heterogeneity: Tau² = 0.00; Chi² = 9.36, df = 16 (P = 0.90); I² = 0%								
Test for overall effect: Z = 6.04 (P < 0.00001)								
**Total (95% CI)**			**439**			**454**	**100.0%**	**7.03 [5.45, 8.61]**
Heterogeneity: Tau² = 6.51; Chi² = 50.78, df = 31 (P = 0.01); I² = 39%								
Test for overall effect: Z = 8.74 (P < 0.00001)								
Test for subgroup differences: Chi² = 3.53, df = 1 (P = 0.06), I² = 71.7%								

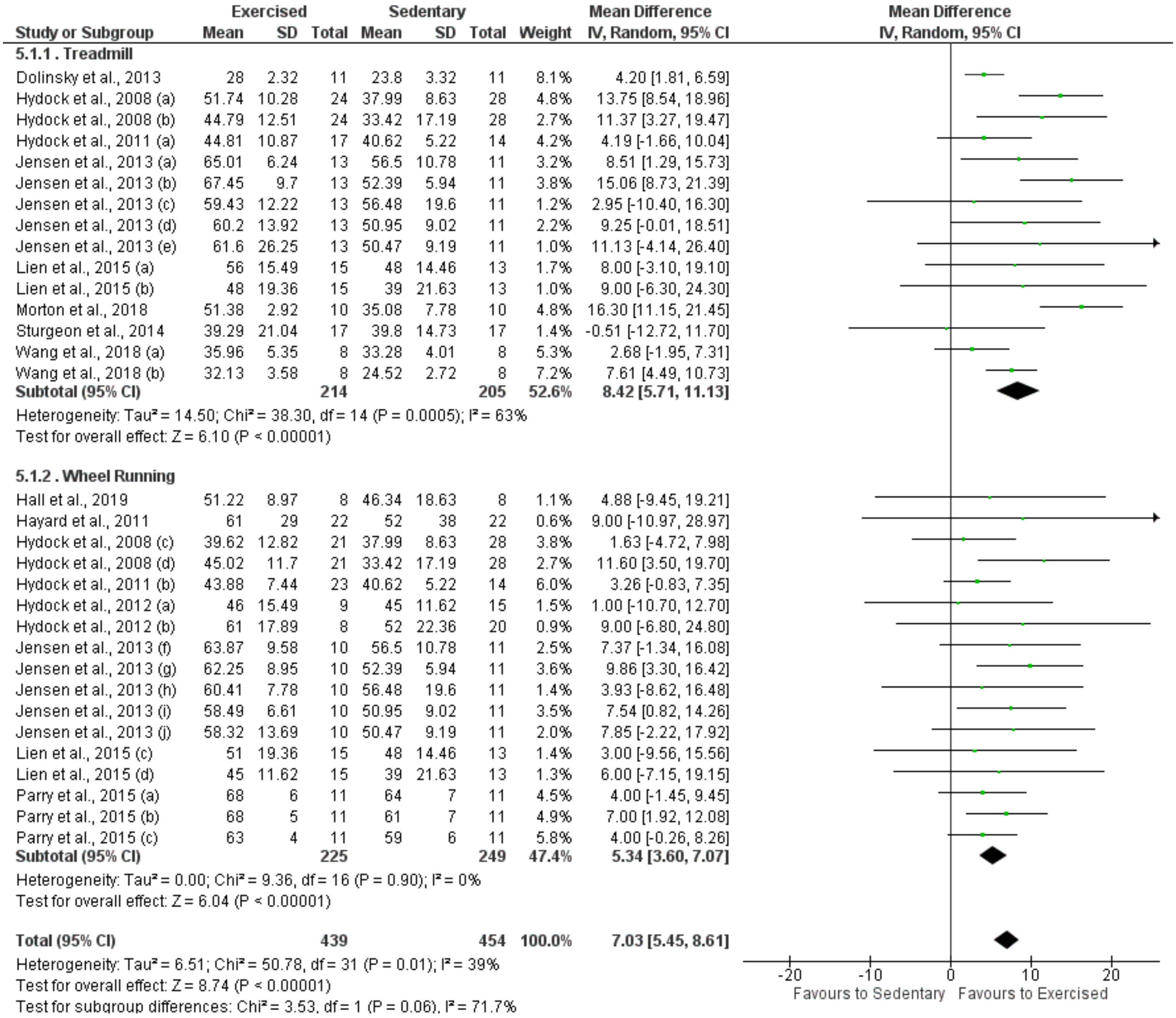

Supplementary Figure S1 – Subgroup analyses of the modality of training protocol (treadmill running vs. voluntary wheel running) on fractional shortening of animals with doxorubicin-induced cardiomyopathy.

Supplementary Figure S2

Study or Subgroup	Exercised Mean	Exercised SD	Exercised Total	Sedentary Mean	Sedentary SD	Sedentary Total	Weight	Mean Difference IV, Random, 95% CI
**4.1.1 . 5 days - 8 weeks**								
Dolinsky et al., 2013	28	2.32	11	23.8	3.32	11	6.8%	4.20 [1.81, 6.59]
Lien et al., 2015 (a)	56	15.49	15	48	14.46	13	1.7%	8.00 [-3.10, 19.10]
Lien et al., 2015 (b)	48	19.36	15	39	21.63	13	1.0%	9.00 [-6.30, 24.30]
Lien et al., 2015 (c)	51	19.36	15	48	14.46	13	1.4%	3.00 [-9.56, 15.56]
Lien et al., 2015 (d)	45	11.62	15	39	21.63	13	1.3%	6.00 [-7.15, 19.15]
Morton et al., 2018	51.38	2.92	10	35.08	7.78	10	4.5%	16.30 [11.15, 21.45]
Sturgeon et al., 2014	39.29	21.04	17	39.8	14.73	17	1.5%	-0.51 [-12.72, 11.70]
Wang et al., 2018 (a)	35.96	5.35	8	33.28	4.01	8	4.9%	2.68 [-1.95, 7.31]
Wang et al., 2018 (b)	32.13	3.58	8	24.52	2.72	8	6.2%	7.61 [4.49, 10.73]
**Subtotal (95% CI)**			**114**			**106**	**29.2%**	**6.68 [3.24, 10.11]**
Heterogeneity: Tau² = 13.15; Chi² = 22.09, df = 8 (P = 0.005); I² = 64%								
Test for overall effect: Z = 3.81 (P = 0.0001)								
**4.1.2 . 10 weeks - 16 weeks**								
Hall et al., 2019	51.22	8.97	8	46.34	18.63	8	1.1%	4.88 [-9.45, 19.21]
Hayard et al., 2011	61	29	22	52	38	22	0.6%	9.00 [-10.97, 28.97]
Hydock et al., 2008 (a)	51.74	10.28	24	37.99	8.63	28	4.4%	13.75 [8.54, 18.96]
Hydock et al., 2008 (b)	44.79	12.51	24	33.42	17.19	28	2.7%	11.37 [3.27, 19.47]
Hydock et al., 2008 (c)	39.62	12.82	21	37.99	8.63	28	3.6%	1.63 [-4.72, 7.98]
Hydock et al., 2008 (d)	45.02	11.7	21	33.42	17.19	28	2.7%	11.60 [3.50, 19.70]
Hydock et al., 2011 (a)	44.81	10.87	17	40.62	5.22	14	4.0%	4.19 [-1.66, 10.04]
Hydock et al., 2011 (b)	43.88	7.44	23	40.62	5.22	14	5.3%	3.26 [-0.83, 7.35]
Hydock et al., 2012 (a)	46	15.49	9	45	11.62	15	1.6%	1.00 [-10.70, 12.70]
Hydock et al., 2012 (b)	61	17.89	8	52	22.36	20	1.0%	9.00 [-6.80, 24.80]
Jensen et al., 2013 (a)	65.01	6.24	13	56.5	10.78	11	3.1%	8.51 [1.29, 15.73]
Jensen et al., 2013 (b)	67.45	9.7	13	52.39	5.94	11	3.6%	15.06 [8.73, 21.39]
Jensen et al., 2013 (c)	59.43	12.22	13	56.48	19.6	11	1.3%	2.95 [-10.40, 16.30]
Jensen et al., 2013 (d)	60.2	13.92	13	50.95	9.02	11	2.3%	9.25 [-0.01, 18.51]
Jensen et al., 2013 (e)	61.6	26.25	13	50.47	9.19	11	1.0%	11.13 [-4.14, 26.40]
Jensen et al., 2013 (f)	63.87	9.58	10	56.5	10.78	11	2.5%	7.37 [-1.34, 16.08]
Jensen et al., 2013 (g)	62.25	8.95	10	52.39	5.94	11	3.5%	9.86 [3.30, 16.42]
Jensen et al., 2013 (h)	60.41	7.78	10	56.48	19.6	11	1.4%	3.93 [-8.62, 16.48]
Jensen et al., 2013 (i)	58.49	6.61	10	50.95	9.02	11	3.4%	7.54 [0.82, 14.26]
Jensen et al., 2013 (j)	58.32	13.69	10	50.47	9.19	11	2.0%	7.85 [-2.22, 17.92]
Parry et al., 2015 (a)	68	6	11	64	7	11	4.2%	4.00 [-1.45, 9.45]
Parry et al., 2015 (b)	68	5	11	61	7	11	4.5%	7.00 [1.92, 12.08]
Parry et al., 2015 (c)	63	4	11	59	6	11	5.2%	4.00 [-0.26, 8.26]
Pfannenstiel et al., 2018	60	6	15	47	4	15	5.7%	13.00 [9.35, 16.65]
**Subtotal (95% CI)**			**340**			**363**	**70.8%**	**7.70 [5.82, 9.58]**
Heterogeneity: Tau² = 7.41; Chi² = 37.37, df = 23 (P = 0.03); I² = 38%								
Test for overall effect: Z = 8.04 (P < 0.00001)								
**Total (95% CI)**			**454**			**469**	**100.0%**	**7.40 [5.75, 9.05]**
Heterogeneity: Tau² = 8.96; Chi² = 61.78, df = 32 (P = 0.001); I² = 48%								
Test for overall effect: Z = 8.80 (P < 0.00001)								
Test for subgroup differences: Chi² = 0.26, df = 1 (P = 0.61), I² = 0%								

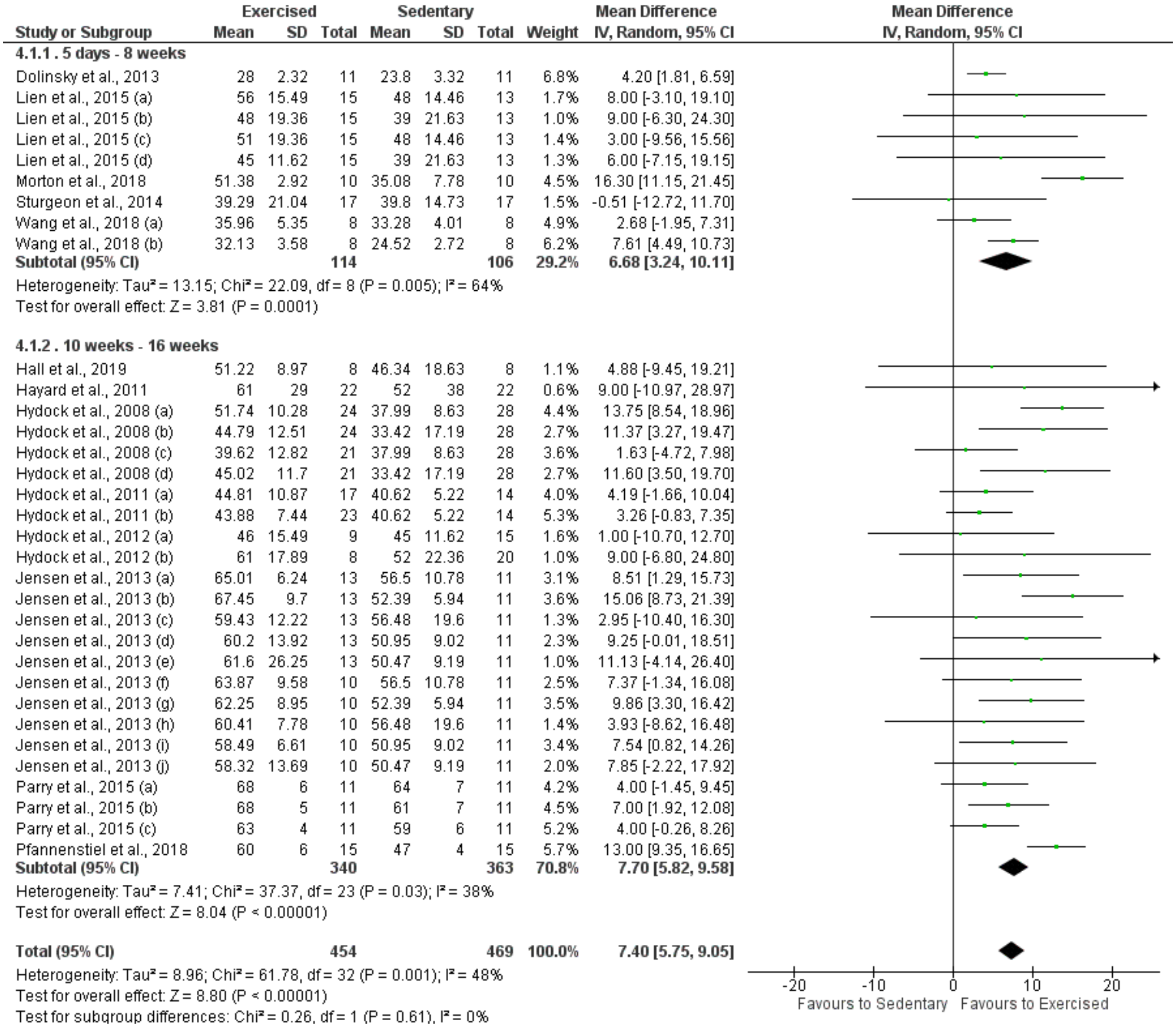

Supplementary Figure S2 – Subgroup analyses of the duration of exercise protocol (5 days to 8 weeks vs. 10 weeks to 16 weeks) on fractional shortening of animals with doxorubicin-induced cardiomyopathy.

Supplementary Figure S3

Study or Subgroup	Exercised Mean	Exercised SD	Exercised Total	Sedentary Mean	Sedentary SD	Sedentary Total	Weight	Mean Difference IV, Random, 95% CI
**1.1.1 . Male Only**								
Hayard et al., 2011	61	29	22	52	38	22	0.6%	9.00 [-10.97, 28.97]
Hydock et al., 2008 (a)	51.74	10.28	24	37.99	8.63	28	4.7%	13.75 [8.54, 18.96]
Hydock et al., 2008 (b)	44.79	12.51	24	33.42	17.19	28	2.8%	11.37 [3.27, 19.47]
Hydock et al., 2008 (c)	39.62	12.82	21	37.99	8.63	28	3.8%	1.63 [-4.72, 7.98]
Hydock et al., 2008 (d)	45.02	11.7	21	33.42	17.19	28	2.8%	11.60 [3.50, 19.70]
Hydock et al., 2011 (a)	44.81	10.87	17	40.62	5.22	14	4.2%	4.19 [-1.66, 10.04]
Hydock et al., 2011 (b)	43.88	7.44	23	40.62	5.22	14	5.6%	3.26 [-0.83, 7.35]
Lien et al., 2015 (a)	56	15.49	15	48	14.46	13	1.8%	8.00 [-3.10, 19.10]
Lien et al., 2015 (b)	48	19.36	15	39	21.63	13	1.0%	9.00 [-6.30, 24.30]
Lien et al., 2015 (c)	51	19.36	15	48	14.46	13	1.5%	3.00 [-9.56, 15.56]
Lien et al., 2015 (d)	45	11.62	15	39	21.63	13	1.4%	6.00 [-7.15, 19.15]
Pfannenstiel et al., 2018	60	6	15	47	4	15	6.0%	13.00 [9.35, 16.65]
Sturgeon et al., 2014	39.29	21.04	17	39.8	14.73	17	1.5%	-0.51 [-12.72, 11.70]
Wang et al., 2018 (b)	32.13	3.58	8	24.52	2.72	8	6.5%	7.61 [4.49, 10.73]
**Subtotal (95% CI)**			**252**			**254**	**44.4%**	**7.64 [4.99, 10.30]**
Heterogeneity: Tau² = 10.63; Chi² = 26.63, df = 13 (P = 0.01); I² = 51%								
Test for overall effect: Z = 5.64 (P < 0.00001)								
**1.1.2 . Female Only**								
Dolinsky et al., 2013	28	2.32	11	23.8	3.32	11	7.2%	4.20 [1.81, 6.59]
Hall et al., 2019	51.22	8.97	8	46.34	18.63	8	1.2%	4.88 [-9.45, 19.21]
Hydock et al., 2012 (a)	46	15.49	9	45	11.62	15	1.6%	1.00 [-10.70, 12.70]
Hydock et al., 2012 (b)	61	17.89	8	52	22.36	20	1.0%	9.00 [-6.80, 24.80]
Jensen et al., 2013 (a)	65.01	6.24	13	56.5	10.78	11	3.3%	8.51 [1.29, 15.73]
Jensen et al., 2013 (b)	67.45	9.7	13	52.39	5.94	11	3.8%	15.06 [8.73, 21.39]
Jensen et al., 2013 (c)	59.43	12.22	13	56.48	19.6	11	1.3%	2.95 [-10.40, 16.30]
Jensen et al., 2013 (d)	60.2	13.92	13	50.95	9.02	11	2.4%	9.25 [-0.01, 18.51]
Jensen et al., 2013 (e)	61.6	26.25	13	50.47	9.19	11	1.1%	11.13 [-4.14, 26.40]
Jensen et al., 2013 (f)	63.87	9.58	10	56.5	10.78	11	2.6%	7.37 [-1.34, 16.08]
Jensen et al., 2013 (g)	62.25	8.95	10	52.39	5.94	11	3.7%	9.86 [3.30, 16.42]
Jensen et al., 2013 (h)	60.41	7.78	10	56.48	19.6	11	1.5%	3.93 [-8.62, 16.48]
Jensen et al., 2013 (i)	58.49	6.61	10	50.95	9.02	11	3.6%	7.54 [0.82, 14.26]
Jensen et al., 2013 (j)	58.32	13.69	10	50.47	9.19	11	2.1%	7.85 [-2.22, 17.92]
Morton et al., 2018	51.38	2.92	10	35.08	7.78	10	4.7%	16.30 [11.15, 21.45]
Parry et al., 2015 (a)	68	6	11	64	7	11	4.5%	4.00 [-1.45, 9.45]
Parry et al., 2015 (b)	68	5	11	61	7	11	4.8%	7.00 [1.92, 12.08]
Parry et al., 2015 (c)	63	4	11	59	6	11	5.5%	4.00 [-0.26, 8.26]
**Subtotal (95% CI)**			**194**			**207**	**55.6%**	**7.61 [5.39, 9.83]**
Heterogeneity: Tau² = 8.24; Chi² = 30.15, df = 17 (P = 0.03); I² = 44%								
Test for overall effect: Z = 6.71 (P < 0.00001)								
**Total (95% CI)**			**446**			**461**	**100.0%**	**7.64 [5.97, 9.32]**
Heterogeneity: Tau² = 8.64; Chi² = 58.14, df = 31 (P = 0.002); I² = 47%								
Test for overall effect: Z = 8.94 (P < 0.00001)								
Test for subgroup differences: Chi² = 0.00, df = 1 (P = 0.98), I² = 0%								

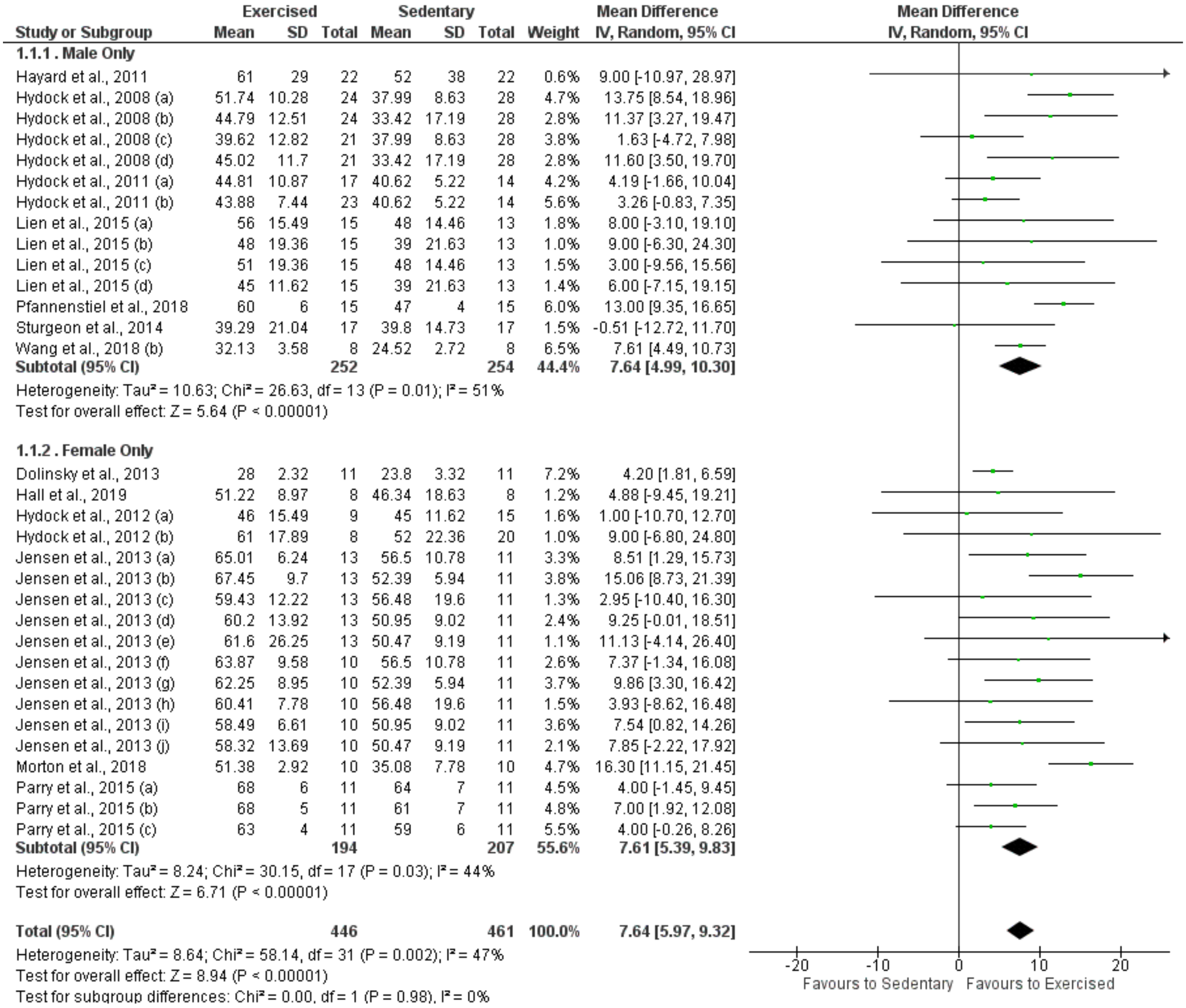

Supplementary Figure S3 – Subgroup analyses of effect the animal gender (male vs. female) on fractional shortening of animals with doxorubicin-induced cardiomyopathy.

Supplementary Figure S4

Study or Subgroup	Exercised Mean	Exercised SD	Exercised Total	Sedentary Mean	Sedentary SD	Sedentary Total	Weight	Mean Difference IV, Random, 95% CI
**3.1.1 . Doxorubicin ≤ 10 mg/Kg**								
Hydock et al., 2008 (a)	51.74	10.28	24	37.99	8.63	28	4.4%	13.75 [8.54, 18.96]
Hydock et al., 2008 (b)	44.79	12.51	24	33.42	17.19	28	2.7%	11.37 [3.27, 19.47]
Hydock et al., 2008 (c)	39.62	12.82	21	37.99	8.63	28	3.6%	1.63 [-4.72, 7.98]
Hydock et al., 2008 (d)	45.02	11.7	21	33.42	17.19	28	2.7%	11.60 [3.50, 19.70]
Hydock et al., 2011 (a)	44.81	10.87	17	40.62	5.22	14	4.0%	4.19 [-1.66, 10.04]
Hydock et al., 2011 (b)	43.88	7.44	23	40.62	5.22	14	5.3%	3.26 [-0.83, 7.35]
Jensen et al., 2013 (a)	65.01	6.24	13	56.5	10.78	11	3.1%	8.51 [1.29, 15.73]
Jensen et al., 2013 (b)	67.45	9.7	13	52.39	5.94	11	3.6%	15.06 [8.73, 21.39]
Jensen et al., 2013 (c)	59.43	12.22	13	56.48	19.6	11	1.3%	2.95 [-10.40, 16.30]
Jensen et al., 2013 (d)	60.2	13.92	13	50.95	9.02	11	2.3%	9.25 [-0.01, 18.51]
Jensen et al., 2013 (e)	61.6	26.25	13	50.47	9.19	11	1.0%	11.13 [-4.14, 26.40]
Jensen et al., 2013 (f)	63.87	9.58	10	56.5	10.78	11	2.5%	7.37 [-1.34, 16.08]
Jensen et al., 2013 (g)	62.25	8.95	10	52.39	5.94	11	3.5%	9.86 [3.30, 16.42]
Jensen et al., 2013 (h)	60.41	7.78	10	56.48	19.6	11	1.4%	3.93 [-8.62, 16.48]
Jensen et al., 2013 (i)	58.49	6.61	10	50.95	9.02	11	3.4%	7.54 [0.82, 14.26]
Jensen et al., 2013 (j)	58.32	13.69	10	50.47	9.19	11	2.0%	7.85 [-2.22, 17.92]
Lien et al., 2015 (a)	56	15.49	15	48	14.46	13	1.7%	8.00 [-3.10, 19.10]
Lien et al., 2015 (c)	51	19.36	15	48	14.46	13	1.4%	3.00 [-9.56, 15.56]
Sturgeon et al., 2014	39.29	21.04	17	39.8	14.73	17	1.5%	-0.51 [-12.72, 11.70]
Wang et al., 2018 (a)	35.96	5.35	8	33.28	4.01	8	4.9%	2.68 [-1.95, 7.31]
Wang et al., 2018 (b)	32.13	3.58	8	24.52	2.72	8	6.2%	7.61 [4.49, 10.73]
**Subtotal (95% CI)**			**308**			**309**	**62.5%**	**7.28 [5.41, 9.16]**
Heterogeneity: Tau² = 5.39; Chi² = 29.26, df = 20 (P = 0.08); I² = 32%								
Test for overall effect: Z = 7.61 (P < 0.00001)								
**3.1.2 . Doxorubicin > 10 mg/Kg**								
Dolinsky et al., 2013	28	2.32	11	23.8	3.32	11	6.8%	4.20 [1.81, 6.59]
Hall et al., 2019	51.22	8.97	8	46.34	18.63	8	1.1%	4.88 [-9.45, 19.21]
Hayard et al., 2011	61	29	22	52	38	22	0.6%	9.00 [-10.97, 28.97]
Hydock et al., 2012 (a)	46	15.49	9	45	11.62	15	1.6%	1.00 [-10.70, 12.70]
Hydock et al., 2012 (b)	61	17.89	8	52	22.36	20	1.0%	9.00 [-6.80, 24.80]
Lien et al., 2015 (b)	48	19.36	15	39	21.63	13	1.0%	9.00 [-6.30, 24.30]
Lien et al., 2015 (d)	45	11.62	15	39	21.63	13	1.3%	6.00 [-7.15, 19.15]
Morton et al., 2018	51.38	2.92	10	35.08	7.78	10	4.5%	16.30 [11.15, 21.45]
Parry et al., 2015 (a)	68	6	11	64	7	11	4.2%	4.00 [-1.45, 9.45]
Parry et al., 2015 (b)	68	5	11	61	7	11	4.5%	7.00 [1.92, 12.08]
Parry et al., 2015 (c)	63	4	11	59	6	11	5.2%	4.00 [-0.26, 8.26]
Pfannenstiel et al., 2018	60	6	15	47	4	15	5.7%	13.00 [9.35, 16.65]
**Subtotal (95% CI)**			**146**			**160**	**37.5%**	**7.50 [4.27, 10.74]**
Heterogeneity: Tau² = 16.20; Chi² = 32.52, df = 11 (P = 0.0006); I² = 66%								
Test for overall effect: Z = 4.55 (P < 0.00001)								
**Total (95% CI)**			**454**			**469**	**100.0%**	**7.40 [5.75, 9.05]**
Heterogeneity: Tau² = 8.96; Chi² = 61.78, df = 32 (P = 0.001); I² = 48%								
Test for overall effect: Z = 8.80 (P < 0.00001)								
Test for subgroup differences: Chi² = 0.01, df = 1 (P = 0.91), I² = 0%								

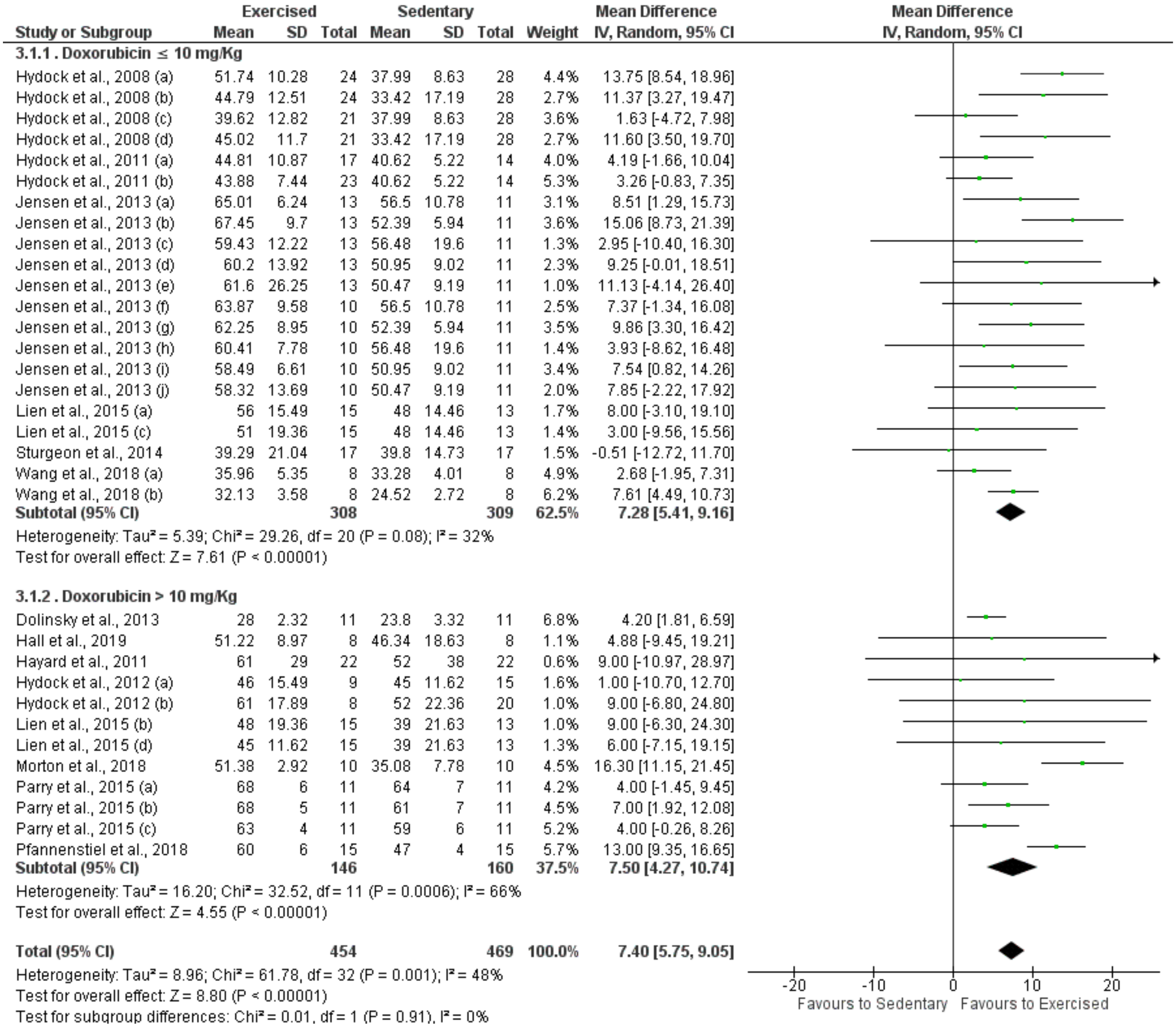

Supplementary Figure S4 – Subgroup analyses of effect the cumulative dose of doxorubicin (≤10 mg/kg vs. >10 mg/kg) on fractional shortening of animals with doxorubicin-induced cardiomyopathy.

Supplementary Figure S5

Study or Subgroup	Exercised Mean	Exercised SD	Exercised Total	Sedentary Mean	Sedentary SD	Sedentary Total	Weight	Mean Difference IV, Random, 95% CI
**7.1.1 . ≤ 10 days**								
Dolinsky et al., 2013	28	2.32	11	23.8	3.32	11	6.8%	4.20 [1.81, 6.59]
Hall et al., 2019	51.22	8.97	8	46.34	18.63	8	1.1%	4.88 [-9.45, 19.21]
Hayard et al., 2011	61	29	22	52	38	22	0.6%	9.00 [-10.97, 28.97]
Hydock et al., 2008 (a)	51.74	10.28	24	37.99	8.63	28	4.4%	13.75 [8.54, 18.96]
Hydock et al., 2008 (b)	44.79	12.51	24	33.42	17.19	28	2.7%	11.37 [3.27, 19.47]
Hydock et al., 2008 (c)	39.62	12.82	21	37.99	8.63	28	3.6%	1.63 [-4.72, 7.98]
Hydock et al., 2008 (d)	45.02	11.7	21	33.42	17.19	28	2.7%	11.60 [3.50, 19.70]
Jensen et al., 2013 (a)	65.01	6.24	13	56.5	10.78	11	3.1%	8.51 [1.29, 15.73]
Jensen et al., 2013 (b)	67.45	9.7	13	52.39	5.94	11	3.6%	15.06 [8.73, 21.39]
Jensen et al., 2013 (c)	59.43	12.22	13	56.48	19.6	11	1.3%	2.95 [-10.40, 16.30]
Jensen et al., 2013 (d)	60.2	13.92	13	50.95	9.02	11	2.3%	9.25 [-0.01, 18.51]
Jensen et al., 2013 (e)	61.6	26.25	13	50.47	9.19	11	1.0%	11.13 [-4.14, 26.40]
Jensen et al., 2013 (f)	63.87	9.58	10	56.5	10.78	11	2.5%	7.37 [-1.34, 16.08]
Jensen et al., 2013 (g)	62.25	8.95	10	52.39	5.94	11	3.5%	9.86 [3.30, 16.42]
Jensen et al., 2013 (h)	60.41	7.78	10	56.48	19.6	11	1.4%	3.93 [-8.62, 16.48]
Jensen et al., 2013 (i)	58.49	6.61	10	50.95	9.02	11	3.4%	7.54 [0.82, 14.26]
Jensen et al., 2013 (j)	58.32	13.69	10	50.47	9.19	11	2.0%	7.85 [-2.22, 17.92]
Lien et al., 2015 (a)	56	15.49	15	48	14.46	13	1.7%	8.00 [-3.10, 19.10]
Lien et al., 2015 (b)	48	19.36	15	39	21.63	13	1.0%	9.00 [-6.30, 24.30]
Lien et al., 2015 (c)	51	19.36	15	48	14.46	13	1.4%	3.00 [-9.56, 15.56]
Lien et al., 2015 (d)	45	11.62	15	39	21.63	13	1.3%	6.00 [-7.15, 19.15]
Morton et al., 2018	51.38	2.92	10	35.08	7.78	10	4.5%	16.30 [11.15, 21.45]
Parry et al., 2015 (a)	68	6	11	64	7	11	4.2%	4.00 [-1.45, 9.45]
Parry et al., 2015 (b)	68	5	11	61	7	11	4.5%	7.00 [1.92, 12.08]
Parry et al., 2015 (c)	63	4	11	59	6	11	5.2%	4.00 [-0.26, 8.26]
Pfannenstiel et al., 2018	60	6	15	47	4	15	5.7%	13.00 [9.35, 16.65]
Sturgeon et al., 2014	39.29	21.04	17	39.8	14.73	17	1.5%	-0.51 [-12.72, 11.70]
Wang et al., 2018 (a)	35.96	5.35	8	33.28	4.01	8	4.9%	2.68 [-1.95, 7.31]
Wang et al., 2018 (b)	32.13	3.58	8	24.52	2.72	8	6.2%	7.61 [4.49, 10.73]
**Subtotal (95% CI)**			**397**			**406**	**88.2%**	**7.89 [6.11, 9.67]**
Heterogeneity: Tau² = 9.51; Chi² = 55.85, df = 28 (P = 0.001); I² = 50%								
Test for overall effect: Z = 8.68 (P < 0.00001)								
**7.1.2 . > 4 weeks**								
Hydock et al., 2011 (a)	44.81	10.87	17	40.62	5.22	14	4.0%	4.19 [-1.66, 10.04]
Hydock et al., 2011 (b)	43.88	7.44	23	40.62	5.22	14	5.3%	3.26 [-0.83, 7.35]
Hydock et al., 2012 (a)	46	15.49	9	45	11.62	15	1.6%	1.00 [-10.70, 12.70]
Hydock et al., 2012 (b)	61	17.89	8	52	22.36	20	1.0%	9.00 [-6.80, 24.80]
**Subtotal (95% CI)**			**57**			**63**	**11.8%**	**3.60 [0.44, 6.75]**
Heterogeneity: Tau² = 0.00; Chi² = 0.70, df = 3 (P = 0.87); I² = 0%								
Test for overall effect: Z = 2.23 (P = 0.03)								
**Total (95% CI)**			**454**			**469**	**100.0%**	**7.40 [5.75, 9.05]**
Heterogeneity: Tau² = 8.96; Chi² = 61.78, df = 32 (P = 0.001); I² = 48%								
Test for overall effect: Z = 8.80 (P < 0.00001)								
Test for subgroup differences: Chi² = 5.40, df = 1 (P = 0.02), I² = 81.5%								

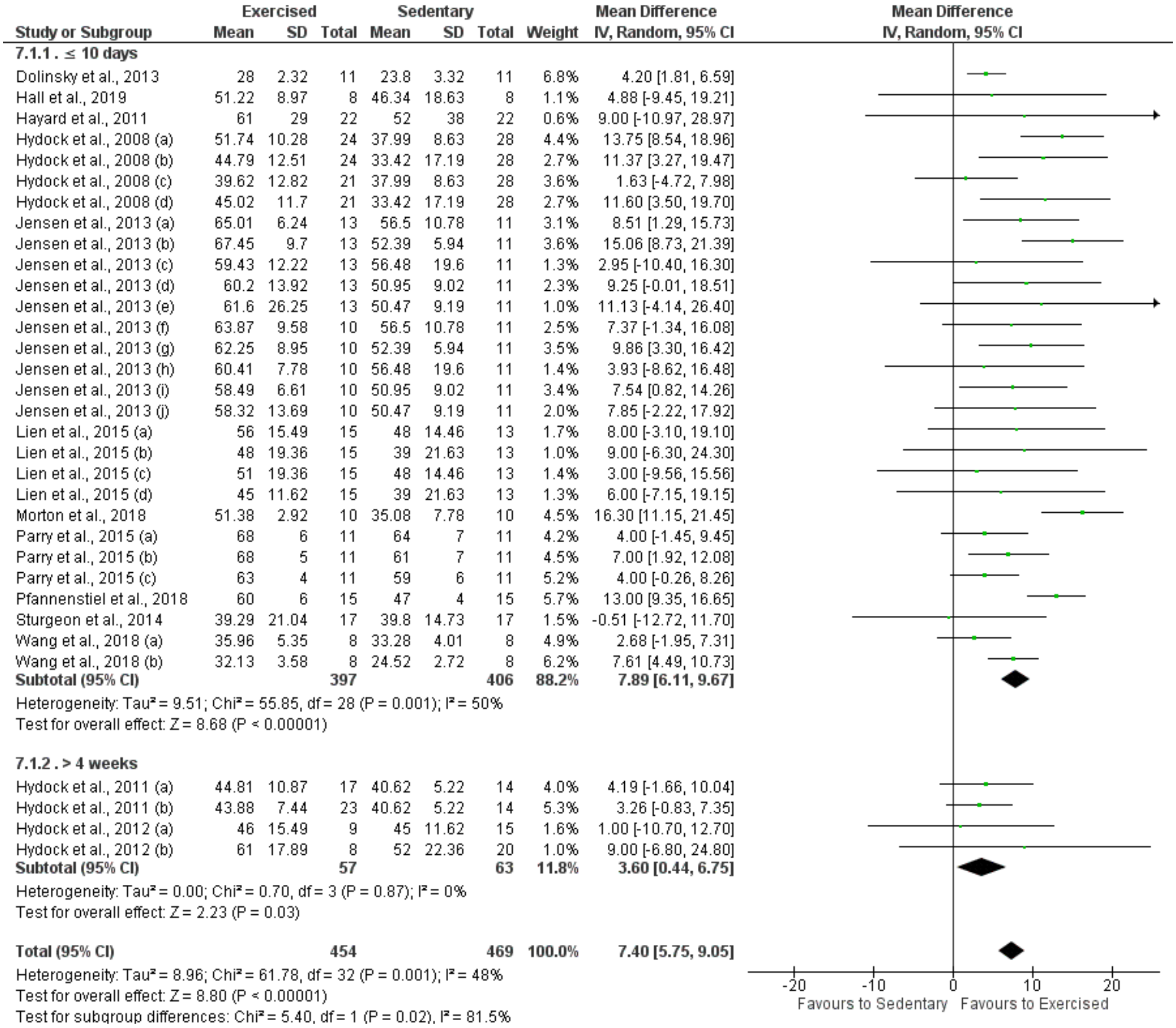

Supplementary Figure S5 – Subgroup analyses of effect the timing of final cardiac function assessment by echocardiography (≤10 days vs. >4 weeks after the end of doxorubicin + exercise training protocol) on fractional shortening of animals with doxorubicin-induced cardiomyopathy.

Supplementary Figure S6

Study or Subgroup	Exercised Mean	Exercised SD	Exercised Total	Sedentary Mean	Sedentary SD	Sedentary Total	Weight	Mean Difference IV, Random, 95% CI
**8.1.1 . Score 5 or 6**								
Hydock et al., 2008 (a)	51.74	10.28	24	37.99	8.63	28	4.4%	13.75 [8.54, 18.96]
Hydock et al., 2008 (b)	44.79	12.51	24	33.42	17.19	28	2.7%	11.37 [3.27, 19.47]
Hydock et al., 2008 (c)	39.62	12.82	21	37.99	8.63	28	3.6%	1.63 [-4.72, 7.98]
Hydock et al., 2008 (d)	45.02	11.7	21	33.42	17.19	28	2.7%	11.60 [3.50, 19.70]
Hydock et al., 2011 (a)	44.81	10.87	17	40.62	5.22	14	4.0%	4.19 [-1.66, 10.04]
Hydock et al., 2011 (b)	43.88	7.44	23	40.62	5.22	14	5.3%	3.26 [-0.83, 7.35]
Hydock et al., 2012 (a)	46	15.49	9	45	11.62	15	1.6%	1.00 [-10.70, 12.70]
Hydock et al., 2012 (b)	61	17.89	8	52	22.36	20	1.0%	9.00 [-6.80, 24.80]
Lien et al., 2015 (a)	56	15.49	15	48	14.46	13	1.7%	8.00 [-3.10, 19.10]
Lien et al., 2015 (b)	48	19.36	15	39	21.63	13	1.0%	9.00 [-6.30, 24.30]
Lien et al., 2015 (c)	51	19.36	15	48	14.46	13	1.4%	3.00 [-9.56, 15.56]
Lien et al., 2015 (d)	45	11.62	15	39	21.63	13	1.3%	6.00 [-7.15, 19.15]
Morton et al., 2018	51.38	2.92	10	35.08	7.78	10	4.5%	16.30 [11.15, 21.45]
Sturgeon et al., 2014	39.29	21.04	17	39.8	14.73	17	1.5%	-0.51 [-12.72, 11.70]
**Subtotal (95% CI)**			**234**			**254**	**36.7%**	**7.46 [4.13, 10.80]**
Heterogeneity: Tau² = 20.09; Chi² = 30.65, df = 13 (P = 0.004); I² = 58%								
Test for overall effect: Z = 4.39 (P < 0.0001)								
**8.1.2 . Score 7 or 8**								
Dolinsky et al., 2013	28	2.32	11	23.8	3.32	11	6.8%	4.20 [1.81, 6.59]
Hall et al., 2019	51.22	8.97	8	46.34	18.63	8	1.1%	4.88 [-9.45, 19.21]
Hayard et al., 2011	61	29	22	52	38	22	0.6%	9.00 [-10.97, 28.97]
Jensen et al., 2013 (a)	65.01	6.24	13	56.5	10.78	11	3.1%	8.51 [1.29, 15.73]
Jensen et al., 2013 (b)	67.45	9.7	13	52.39	5.94	11	3.6%	15.06 [8.73, 21.39]
Jensen et al., 2013 (c)	59.43	12.22	13	56.48	19.6	11	1.3%	2.95 [-10.40, 16.30]
Jensen et al., 2013 (d)	60.2	13.92	13	50.95	9.02	11	2.3%	9.25 [-0.01, 18.51]
Jensen et al., 2013 (e)	61.6	26.25	13	50.47	9.19	11	1.0%	11.13 [-4.14, 26.40]
Jensen et al., 2013 (f)	63.87	9.58	10	56.5	10.78	11	2.5%	7.37 [-1.34, 16.08]
Jensen et al., 2013 (g)	62.25	8.95	10	52.39	5.94	11	3.5%	9.86 [3.30, 16.42]
Jensen et al., 2013 (h)	60.41	7.78	10	56.48	19.6	11	1.4%	3.93 [-8.62, 16.48]
Jensen et al., 2013 (i)	58.49	6.61	10	50.95	9.02	11	3.4%	7.54 [0.82, 14.26]
Jensen et al., 2013 (j)	58.32	13.69	10	50.47	9.19	11	2.0%	7.85 [-2.22, 17.92]
Parry et al., 2015 (a)	68	6	11	64	7	11	4.2%	4.00 [-1.45, 9.45]
Parry et al., 2015 (b)	68	5	11	61	7	11	4.5%	7.00 [1.92, 12.08]
Parry et al., 2015 (c)	63	4	11	59	6	11	5.2%	4.00 [-0.26, 8.26]
Pfannenstiel et al., 2018	60	6	15	47	4	15	5.7%	13.00 [9.35, 16.65]
Wang et al., 2018 (a)	35.96	5.35	8	33.28	4.01	8	4.9%	2.68 [-1.95, 7.31]
Wang et al., 2018 (b)	32.13	3.58	8	24.52	2.72	8	6.2%	7.61 [4.49, 10.73]
**Subtotal (95% CI)**			**220**			**215**	**63.3%**	**7.21 [5.38, 9.05]**
Heterogeneity: Tau² = 5.52; Chi² = 30.50, df = 18 (P = 0.03); I² = 41%								
Test for overall effect: Z = 7.72 (P < 0.00001)								
**Total (95% CI)**			**454**			**469**	**100.0%**	**7.40 [5.75, 9.05]**
Heterogeneity: Tau² = 8.96; Chi² = 61.78, df = 32 (P = 0.001); I² = 48%								
Test for overall effect: Z = 8.80 (P < 0.00001)								
Test for subgroup differences: Chi² = 0.02, df = 1 (P = 0.90), I² = 0%								

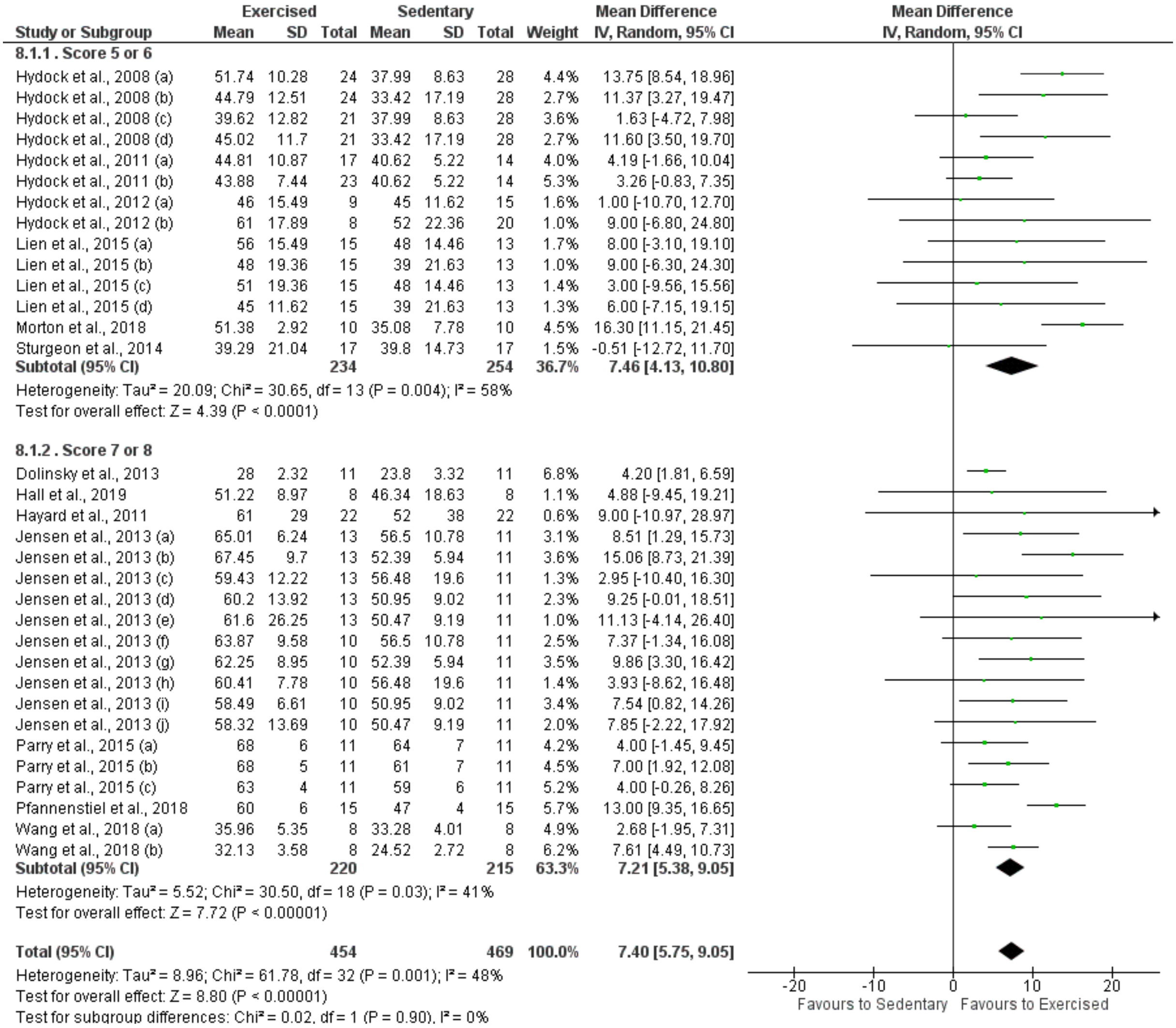

Supplementary Figure S6 – Subgroup analyses of the effect of study quality on fractional shortening of animals with doxorubicin-induced cardiomyopathy according to CAMARADES checklist (score 5 or 6 vs. score 7 or 8

Supplementary Figure S7

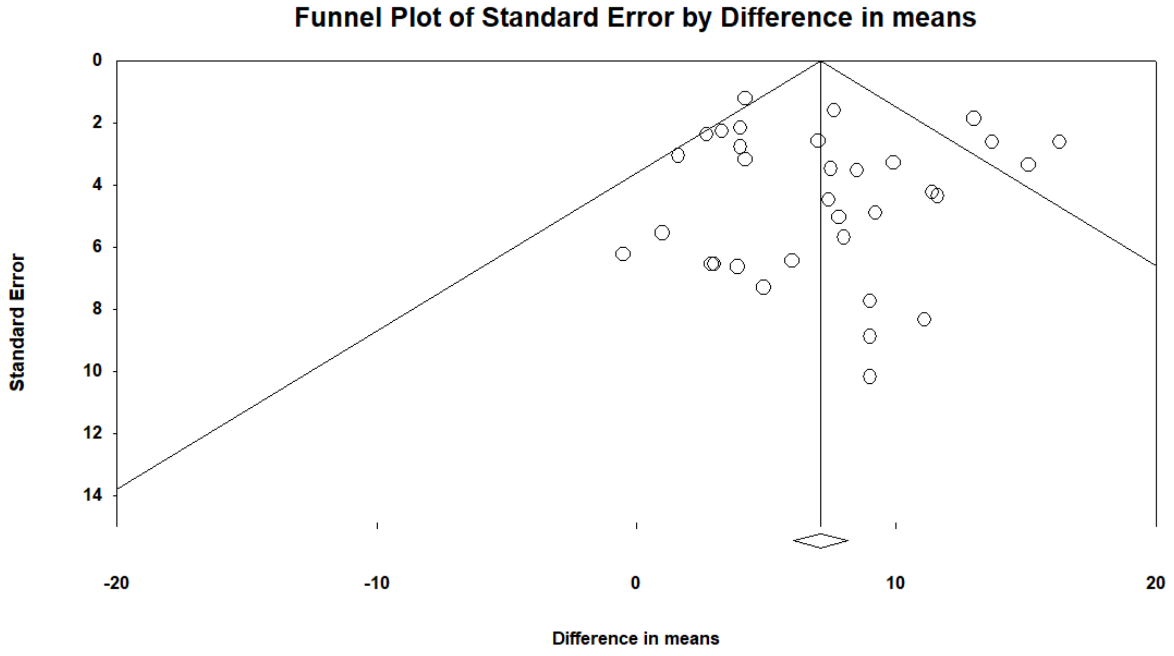

Supplementary Figure S7 – Publication bias: funnel plot for all studies reporting fractional shortening as outcome of cardiac function Egger's regression test, 2-tailed, p=0.675.
